# Formalin-Inactivated EV71 Vaccine Candidate Induced Cross-Neutralizing Antibody against Subgenotypes B1, B4, B5 and C4A in Adult Volunteers

**DOI:** 10.1371/journal.pone.0079783

**Published:** 2013-11-21

**Authors:** Ai-Hsiang Chou, Chia-Chyi Liu, Jui-Yuan Chang, Renee Jiang, Yi-Chin Hsieh, Amanda Tsao, Chien-Long Wu, Ju-Lan Huang, Chang-Phone Fung, Szu-Min Hsieh, Ya-Fang Wang, Jen-Ren Wang, Mei-Hua Hu, Jen-Ron Chiang, Ih-Jen Su, Pele Choi-Sing Chong

**Affiliations:** 1 Vaccine Research & Development Center, National Institute of Infectious Diseases and Vaccinology, National Health Research Institutes, Zhunan Town, Miaoli County, Taiwan; 2 Veterans General Hospital Taipei, Taipei, Taiwan; 3 National Taiwan University Hospital, Taipei, Taiwan; 4 Taiwan CDC, Taipei, Taiwan; 5 Graduate Institute of Immunology, China Medical University, Taichung, Taiwan; National Taiwan University Hospital, Taiwan

## Abstract

**Background:**

Enterovirus 71 (EV71) has caused several epidemics of hand, foot and mouth diseases (HFMD) in Asia. No effective EV71 vaccine is available. A randomized and open-label phase I clinical study registered with ClinicalTrials.gov #NCT01268787, aims to evaluate the safety, reactogenicity and immunogenicity of a formalin-inactivated EV71 vaccine candidate (EV71vac) at 5- and 10-µg doses. In this study we report the cross-neutralizing antibody responses from each volunteer against different subgenotypes of EV71 and CVA16.

**Methods:**

Sixty eligible healthy adults were recruited and vaccinated. Blood samples were obtained on day 0, 21 and 42 and tested against B1, B4, B5, C2, C4A, C4B and CVA16 for cross-neutralizing antibody responses.

**Results:**

The immunogenicity of both 5- and 10- µg doses were found to be very similar. Approximately 45% of the participants had <8 pre-vaccination neutralization titers (Nt) against the B4 vaccine strain. After the first EV71vac immunization, 95% of vaccinees have >4-fold increase in Nt, but there was no further increase in Nt after the second dose. EV71vac induced very strong cross-neutralizing antibody responses in >85% of volunteers without pre-existing Nt against subgenotype B1, B5 and C4A. EV71vac elicited weak cross-neutralizing antibody responses (∼20% of participants) against a C4B and Coxsackie virus A16. Over 90% of vaccinated volunteers did not develop cross-neutralizing antibody responses (Nt<8) against a C2 strain. EV71vac can boost and significantly enhance the neutralizing antibody responses in volunteers who already had pre-vaccination antibodies against EV71 and/or CVA16.

**Conclusion:**

EV71vac is efficient in eliciting cross-neutralizing antibody responses against EV71 subgenotypes B1, B4, B5, and C4A, and provides the rationale for its evaluation in phase II clinical trials.

**Trial Registration:**

ClinicalTrials.gov __NCT01268787

## Introduction

Enterovirus 71 (EV71) infections is the leading cause of hand, foot and mouth disease (HFMD) in Asian children that can result in severe neurological complications and death [1]–[4]. As such, the WHO has identified it as an emerging infectious disease in the Far East (http://www.wpro.who.int/media_centre/news/news_20090713.htm). Therefore, EV71 vaccine is urgently needed, and promising candidates are being evaluated in human phase I clinical trials [5]–[6].

EV71 belongs to a non-enveloped RNA virus of the family *Picornaviridae*, and contains a plus sense ssRNA (7.5–8.5 kb) and four structural proteins: VP1, VP2, VP3 and VP4 [7]–[9]. EV71 is currently classified into 3 genotypes, A, B and C. Genotypes B and C are further divided into B1–B5 and C1–C5 subgenotypes [10]–[11]. B5 isolates were found in recent HFMD epidemics in Malaysia, Singapore, Taiwan, and Thailand, but the C4 genotype found in mainland China [11]–[12]. Therefore, an effective EV71 vaccine should elicit strong cross-neutralizing antibody responses against different EV71 genotypes.

**Figure 1:**
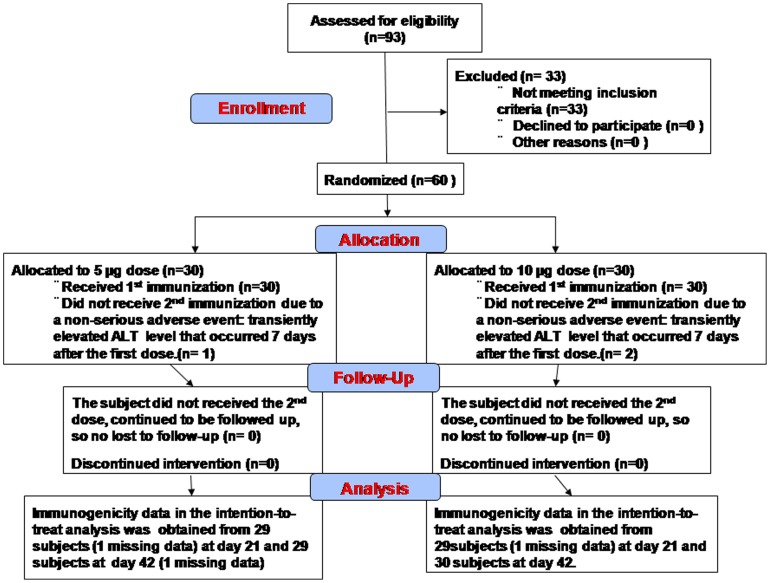
The CONSORT flowchart of EV71vac phase 1 clinical trial. The detail of volunteer enrollment is now fully described in our previous report [24].

The inactivated EV71 virion produced in Vero cells has been shown to elicit more efficacious immune responses than those obtained from recombinant VP1 protein or DNA vector vaccines in the mouse challenge model [3]–[6], [13]–[23]. A vaccine strain based on the EV71 B4 subgenotype (EV71/E59) strain was selected and fully characterized according to US FDA guidelines [5], [20]. To complete the regulatory dossier, we published a series of peer-reviewed papers describing a scalable and reliable manufacturing process for a chemically-inactivated EV71 vaccine candidate (EV71vac) that was found to be capable of eliciting cross-neutralizing antibody responses in different animal immunogenicity studies [5], [9], [18]–[23]. In this study, we report the results of human immunogenicity of EV71vac at 5- and 10-µg doses in 60 healthy volunteers according to NHRI clinical protocol #QCR10013 (Figure 1). This study is registered with ClinicalTrials.gov with the registration #NCT01268787. The protocol was approved by Taiwan FDA #9917719 and conducted in compliance with National Taiwan University Hospital (NTUH) IRB# 201007056M and Veteran General Hospital Taipei IRB#201008015MA. The inclusion/exclusion criteria for the subject recruitment and randomization were reported in our recent publication [24].

## Materials and Methods

The protocol for this trial and supporting CONSORT checklist are available as supporting information; see Checklist S1 and Clinical Trial Protocol S1.

### Ethics Statement

In this study, we report the results of human immunogenicity of EV71vac at 5- and 10-µg doses in 60 healthy volunteers according to NHRI clinical protocol #QCR10013. This study is registered with ClinicalTrials.gov #NCT01268787. The protocol was approved by Taiwan FDA #9917719 and conducted in compliance with National Taiwan University Hospital (NTUH) IRB# 201007056M and Veteran General Hospital Taipei IRB#201008015MA. The trial was started in December 2010 and completed in March 2012 for patient recruitment and follow-up. At the screening visit (Visit 1), the investigator verbally reviewed the written, informed consent form (ICF, a copy attached in the Supporting Information) with each volunteer to ensure he/she understanding the study and the study procedures. Signed informed consent was obtained before performing any procedures related to the study and after the volunteers had received sufficient information about the study and had had the opportunity to ask any questions and consider the options. A copy of the signed ICF was provided to the subject and the original document retained in the study file.

### Cells and Viruses

All EV71 strains used for *in vitro* neutralization assay were grown in Rhabdomyosarcoma (RD) cells. EV71/E59 and EV71/E36 virus strains were provided by Taiwan CDC [21]. EV71 isolates 0204/TW86 (B1 subgenotype), N0692/TW08 (B5), N3340/TW02 (C2), 5746/TW98 (C4B), and Coxsackie virus A16 5079/TW98 were obtained from National Cheng Kung University.

### EV71 vaccine candidate (EV71vac)

Vero cell banks and EV71 virus seed banks (E59 strain) were established according to US FDA cGMP guidelines as described in previous publications [5], [21]. The EV71 vaccine candidate (EV71vac) was produced and fully characterized as previously reported [5], [9], [21]–[23].

### Phase I clinical study

This clinical study was a phase I, uncontrolled, prospective, randomized, open-label, two-center clinical study in Taipei, Taiwan. The safety, reactogenicity and preliminary immunogenicity of EV71vac at 5- and 10-µg doses in sixty eligible healthy volunteers are currently reported elsewhere [24]. After enrollment, all subjects were randomized into 2 dosing groups in a 1∶1 ratio. Groups A and B were immunized with 0.25 mL per dose (5 µg total protein+adjuvant 150 µg AIPO_4_) and 0.5 mL per dose (10 µg total protein+adjuvant 300 µg AIPO_4_), respectively. Both groups followed the same visit schedule and were vaccinated intramuscularly with one dose of EV71vac on Day 0 and a second dose on Day 21. Serum samples were collected at Day 0, 21 and 42, and inactivated at 56°C for 30 minutes and stored at −80°C before use.

### Virus neutralization assay

The cytopathic effect (CPE) assay was used to evaluate virus neutralization titers (Nt) which was defined as the reciprocal of the highest dilution capable of inhibiting 50% of CPE [5], [25].

### EV71-Specific ELISA

Specific IgG titer against EV71/E59 was determined by enzyme-linked immunosorbent assay (ELISA). Inactivated EV71/E59 (1 g/mL) was first coated onto 96-well microplates and allowed to stand overnight. The microplates were then washed with 0.5% Tween 20 in phosphate buffer saline (PBST) and blocked with 1% bovine serum albumin (BSA) in PBS for 2 hours at room temperature. After washing the microplates with PBST for a second time, 100 µL of two-fold serial diluted clinical serum specimens (beginning with 1∶1000) were added into each well. The microplates were incubated at room temperature for 2 hours, and then washed with PBST. One hundred microliters of the detection antibody (i.e., goat anti-human IgG-Fc antibody conjugated with HRP; 1∶5000 diluted) was added into each well and incubated on the microplates at room temperature for 1 hour. After washing the microplates with PBST, 100 µL the chromogenic substrate 2,2-azino-di-(ethylbenzthiazoline-sulfonic acid) (ABTS) was added into each well and incubated at room temperature for 20 minutes. A spectrophotometer measured the absorbance at 405 nm. The IgG titer was defined as the endpoint of serial dilution at which the optical density (OD) value was two-fold higher than the background value that was obtained from the negative control peptide CB82 with sequence (DVSDFTDSVRDPKTS) derived from SARS S protein.

### Statistical analyses

All statistical analyses were performed using SAS software and all tests were two-sided when applied. Statistical significance for all comparisons was determined at P<0.05.

## Results

### Individual antibody response to EV71vac immunization

One of the enrollment criteria was adult with ages between 20 and 60 years. In this trial, the mean age of the volunteers was found to be 29.3±4.9 and 28.6±5.0 for 5 and 10 microgram dosage groups, respectively. One subject withdrew from the study; and one blood sample was misplaced and lost (Figure 1). The rate of seroconversion was defined as the proportion of subjects with an increase in neutralizing antibody titer by a factor of four or more based the B4 virus neutralization assay [24]. In this study, we carefully analyze the antibody responses of individual volunteer. Ten volunteers (#12, 14, 18, 22, 31, 34, 39, 43, 44 & 54) were found to have low IgG reactivity with EV71 (<1000 titer) and no EV71 virus neutralizing antibodies (VNA) against vaccine strain virus in their pre-immune sera (Table S1 in File S1). They most likely had not been exposed to or infected by EV71. Their IgG titers increased ≥4-fold after one dose of EV71vac (Table S1 in File S1). Only two adults (Subject #15 and #56 received 5- and 10-µg of EV71vac, respectively) with pre-existing anti-EV71 IgG antibodies (4000 titer) did not show a 4-fold increase after two EV71vac doses. The remaining 57 participants all had a ≥4-fold increase in anti-EV71 IgG titers.

Twenty-seven out of sixty participants were found to have <8 Nt against vaccine strain in their pre-immune sera (Table S1 in File S1). A >4-fold increase in Nt after the first vaccination was observed in 92% and 100% of participants from Group A and B who had a pre-immune Nt of <8, respectively (Table 1). After one dose, the geometric mean titers (GMT) were found to be 210.9 and 259.8 for Group A and B, respectively (Table 1). Nine out of ten potential naive subjects (#12, 14, 18, 22, 31, 34, 39, 43, 44, and 54) were found to have their Nt increased ≥4-fold after 1 dose of either 5- or 10-µg of EV71vac (Table S1 in File S1). This indicates that a 5-µg dose of EV71vac is as immunogenic as a 10-µg dose in adult volunteers.

**Table 1:** Summary of EV71 B4 subgenotype-specific virus neutralizing antibody titers in sera from subjects immunized with the 5 and 10 µg of EV71 vaccine as Group A and B, respectively.

Group	Pre-vaccination		>4-fold increased post 1^st^-vaccination	Neutralization titer against B4 (GMT)	>4-fold increased post 2^nd^-vaccination	Neutralization titer against B4 (GMT)
A	<1∶8	13/30 (43.3%)	12/13 (92.3%)	210.9	13/13 (100%)	181.3
	≥1∶8	17/30 (56.7%)	15/17 (88.2%)	606.2	17/17 (100%)	777.9
B	<1∶8	14/30 (46.7%)	13/13* (100%)	259.8	13/14 (92.9%)	175.3
	≥1∶8	16/30 (53.3%)	14/15** (93.3%)	1068.4	14/15** (93.3%)	993.7

⋇ Nt below detection limit (<1∶8) were assigned a value of 1∶4 for calculation purposes.

* One blood sample was misplaced and lost.

** One subject withdrew from the study.

Greater than 90% (29/32) of participants in both groups with pre-existing Nt were found to exhibit a >4-fold increase in Nt after the first vaccination (Table 1). Their GMT of Nt were found to be significantly higher than those obtained from volunteers without pre-existing Nt in both Group A (606.2 vs. 210.9) and B (1068.4 vs. 259.8) (p<0.001 based on student t-test) (Table 1). Essentially all participants in Groups A & B showed a >4-fold increase in Nt after the second vaccination (Table 1). It was a surprise that no further significant increase in Nt was observed after the booster immunization. In fact, the GMT of Nt was found to be decreased in volunteers (Table 1 & Fig. S1 in File S1), but was not statistically significant based on the student t-test (p<0.6).

### Cross-neutralizing antibody responses against different EV71 genotypes

**Figure 2:**
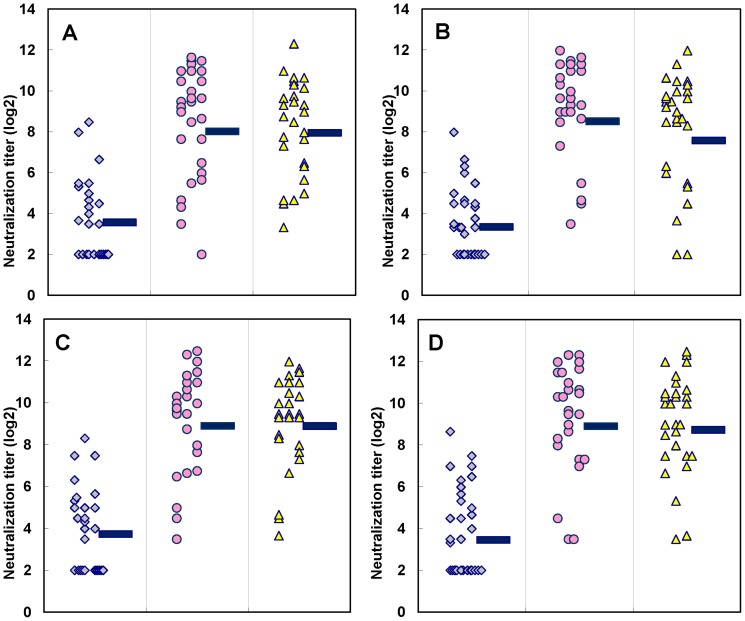
Virus neutralizing antibody responses of adults immunized with EV71vac against subgenotypes B1 and B5. Panels A and B show the B1 neutralization titers of post-vaccination sera obtained with a 5- and 10-µg dose, respectively. Panels C and D show the B5 neutralization titers of post-vaccination sera obtained with a 5- and 10-µg dose, respectively. Each spot represents the neutralizing antibody response for individual serum. The bar corresponds to the geometric mean titer (GMT) of each set of sera.

Careful immunological analyses of the sera showed that 21 participants had no detectable Nt (<8) against EV71 subgenotypes B1 and B5 before vaccination, and that a >4-fold increase in Nt was observed in 17/21 and 18/21 (>80%) of participants after the first vaccination, respectively (Table S2 in File S1 & Fig. 2). Based on the GMT of Nt, most of volunteers with pre-immune Nt <8 show better responses against B5 than B1 (243–265 vs 92–130) (p<0.003 based on student t-test) (Table S2 in File S1). On contrary, most of volunteers with pre-immune Nt>8 show comparable GMT of Nt (about 1000). Their GMT of Nt were found to be significantly higher than those obtained from volunteers without pre-existing Nt in both Group A and B (870–1143 vs. 92–265) (p<0.001 based on student t-test) (Table S2 in File S1). Again, in general the GMT of Nt shows a decrease in Nt against B1 and B5 after the second vaccination (Table S2 in File S1). The current results indicate that a 5-µg dose of EV71vac could elicit strong VNA responses against EV71 subgenotypes B1 and B5 in >80% of tested adults.

Most of participants (57/60) had no detectable Nt against subgenotype C2 before vaccination, and even after two doses of EV71vac only nine of 56 subjects (#2, 4, 6, 9, 10, 16, 19, 30 & 35) in both groups mounted a positive neutralizing response (Table S1 in File S1). Only four of 29 volunteers in the 10-µg dose had >4-fold increased Nt (Table 2). Interestingly, 2 out 3 (67%) of participants with pre-existing Nt displayed a 4-fold increase in Nt after 2×5-µg immunizations (Table 2). These results suggest that sera from EV71vac vaccinees have poor cross-neutralization activity against the EV71 C2 subgenotype.

**Table 2:** Summary of EV71 virus neutralizing antibody titers against EV71 C2 subgenotype and CVA16 obtained from subjects immunized with the 5 and 10 µg of EV71 vaccine as Group A and B, respectively.

			Post 1^st^-vaccination		Post 2^nd^-vaccination	
EV71 virus	Group	Pre-vaccination	>4-fold increase Nt	Neutralization titer (GMT)	>4-fold increase Nt	Neutralization titer (GMT)
	A <1∶8	27/30 (90.0%)	0/27 (0%)	4.6	0/27 (0%)	4.8
EV71/5746/TW98 (C2)	≥1∶8	3/30 (10.0%)	0/3 (0%)	15.3	2/3 (66.7%)	89.1
	B <1∶8	30/30 (100%)	1/28^a^^, ^ b (3.6%)	4.9	4/29^b^ (13.8%)	6.1
	≥1∶8	0/30 (0%)	0/0 (0%)	<4	0/0 (0%)	<4
	A <1∶8	27/30 (90.0%)	7/27 (25.9%)	8.7	7/27 (25.9%)	8.6
CV/5079/TW98 (CVA16)	≥1∶8	3/30 (10.0%)	0/3 (0%)	6.8	0/3 (0%)	12.1
	B <1∶8	22/30 (73.3%)	3/21^a^ (14.3%)	7.5	6/22 (27.3%)	8.2
	≥1∶8	8/30 (26.7%)	1/7^b^ (14.3%)	22.4	2/7 (28.6%)	38.3

Nt below detection limit (<1∶8) were assigned a value of 1∶4 for calculation purposes.

a One blood sample was misplaced and lost.

b One subject withdrew from study.

**Figure 3:**
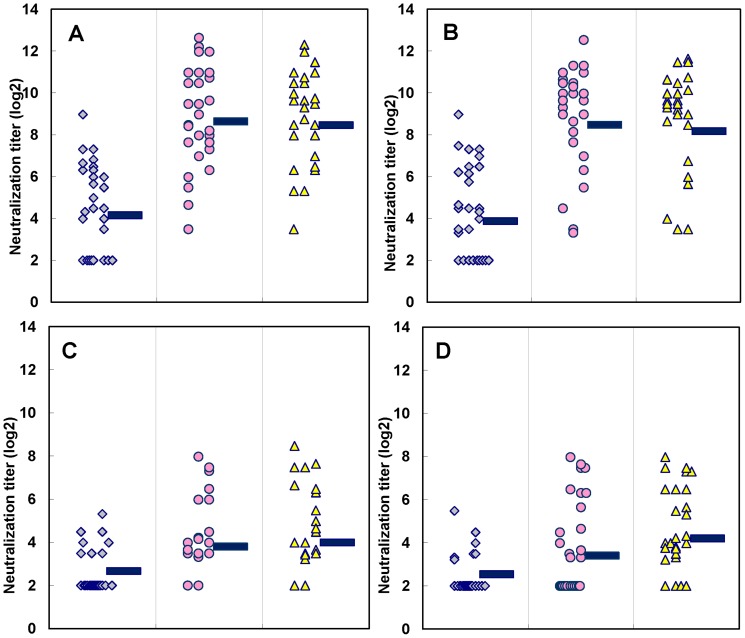
Virus neutralizing antibody responses of adults immunized with EV71vac against subgenotypes C4A and C4B. Panels A and B show the C4A neutralization titers of post-vaccination sera obtained with a 5- and 10-µg dose, respectively. Panels C and D display the C4B neutralization titers of post-vaccination sera obtained with a 5- and 10-µg dose, respectively. Each spot represents the neutralizing antibody response for individual sera. The bar represents the geometric mean titer (GMT) of each set of sera.

The cross-reactivity against genotype C4 was found to be more complicated. Before vaccination, 30% of participants (18/60) had no detectable Nt against the EV71/E36 strain (subgenotype C4A that circulated in Taiwan in 2004) (Fig. 3). After one immunization, >80% (13/16) of these participants raised their Nt >4-fold (Table S3 in File S1). Seventy-five percent (33/42) of participants with pre-vaccination Nt (>8) also had a >4-fold increase in Nt. Again, volunteers with pre-vaccination Nt had higher GMT of Nt than those without pre-existing Nt (>900 vs. 90) (p<0.001 based on student t-test) (Table S3 in File S1). However, when tested against the C4B subgenotype strain (isolated during the 2002 EV71 outbreak in Taiwan), >60% (37/60) of participants had no detectable Nt (<8) against C4B in their pre-immune sera. Although 28% (5/18) and 18% (3/17) of participants with Nt<8 in 5 and 10 µg dosage, respectively had a >4-fold increase in Nt after 1 dose of EV71vac, their GMTs of Nt were significantly lower (10.6 and 8.6 for 5 and 10 µg dosage, respectively) compared to those obtained for subgenotype C4A (98.0 and 74.0 for 5 and 10 µg dosage, respectively) (p<0.001 based on student t-test) (Table S3 in File S1). For participants with pre-vaccination Nt>8, about 54–58% of them mounted a 4-fold increase in Nt against C4B after 2 doses (Table S3 in File S1). Again, their GMTs of Nt were significantly lower (∼90) compared to those obtained from subgenotype C4A (>900) (p<0.001 based on student t-test) (Table S3 in File S1). Therefore, the current results indicate that EV71vac could elicit strong VNA responses against subgenotype C4A, but weak against C4B.

The analysis of virus cross-neutralization data against CVA16 revealed that 49/60 (>80%) of the pre-immune sera had no detectable Nt (<8) (Table S1 in File S1). After the first EV71vac vaccination, 25% (7/27) and 14% (3/21) of these participants in Group A and B, respectively showed a >4-fold increase in Nt regardless of the vaccine dose (Table 2). The GMT of Nt was found to be low (∼8). Only Subject #17 had a >4-fold increase in Nt after two vaccinations, but the Nt was moderate (50) compared to those obtained for B4 (178) (Table S1 in File S1). After 2 doses, vaccinated sera from Group B (10-µg dose) were found to have more subjects (from 14% (1/7) increased to 28% (2/7) with 4-fold increases in the Nt (Table 2). The number of volunteer with 4-fold increase in the Nt against CVA16 after 2 doses in both groups (7/30 in group A and 8/29 in group B) are found to be not much of different (p<0.3 based on student t-test). Thus, the current results indicate a weak VNA response against CVA16 in EV71vac vaccinated adults.

### Immune responses from potential naïve volunteers

As mentioned above, ten subjects had pre-immune Nt<8 and low IgG titers (<1000) against the B4 vaccine strain. Except Subject #18, all produced strong Nt against B4 after one EV71vac immunization (Table 3). In addition, 40% of these 10 volunteers (Subject #14, 34, 39 and 44) were found to have Nt against CVA16 after EV71vac immunizations. These sera were further analyzed against subgenotypes B1, B5, C2, C4A and C4B and confirmed that five subjects (#14, 18, 22, 31 and 39) had most likely not been exposed to or infected with EV71 (Table 3). All these five subjects produced Nt against B4, B1, B5, and C4A subgenotypes after EV71vac immunizations (Table 3). Subjects #12, 34, 43, 44 and 54 were found to have low but positive pre-vaccinated Nt against subgenotype (B1 & C4A), (C4A), (B1, B5, C4A & B), (C4A & B), and (B5, C4A & B), respectively (Table 3). All these five subjects produced strong Nt against B4, B1, B5, C4A and C4B subgenotypes after one EV71vac immunization (Table 3). Surprisingly, no VNA responses against C2 were observed from these ten potential naïve volunteers who had been vaccinated twice (Table 3). In general, those subjects found to have low Nt against EV71 subgenotypes in their pre-vaccinated sera normally produced strong Nt against other genotypes after one EV71vac immunizations (Table 3) (p<0.001 based on student t-test). In Table 3, Subject #12, 34, 39, 43, 44 and 54 were most likely primed with different genotype of EV71, they produced significant neutralizing antibody responses when they were immunized with EV71vac.

**Table 3:** Summary of EV71 subgenotypes-specific virus neutralizing antibody titers obtained from naïve adult sera immunized with the 5 and 10 µg of EV71 vaccine as Group A and B, respectively.

		Pre-vaccination						Post 1^st^-vaccination (Nt)						Post 2^nd^-vaccination (Nt)					
Group	Subject #	B4	B1	B5	C2	C4A	C4B	B4	B1	B5	C2	C4A	C4B	B4	B1	B5	C2	C4A	C4B
	14	<8	<8	<8	<8	<8	<8	40	20	32	<8	63	<8	50	25	25	<8	79	<8
A	18	<8	<8	<8	<8	<8	<8	<8	11	11	<8	11	<8	16	10	13	<8	11	<8
	44	<8	<8	<8	<8	11	11	1419	1419	4019	<8	4019	158	711	632	2010	<8	711	79
	54	<8	<8	16	<8	16	16	1419	798	4019	<8	798	89	399	502	1265	<8	857	89
	12	<8	11	<8	<8	22	<8	1419	502	1264	<8	1005	<8	1004	711	1005	<8	399	<8
	22	<8	<8	<8	<8	<8	<8	40	11	11	<8	11	<8	45	13	40	<8	11	<8
	31	<8	<8	<8	<8	<8	<8	20	11	11	<8	10	<8	<8	<8	11	<8	11	<8
B	34	<8	<8	<8	<8	11	<8	11	2010	2010	<8	2530	<8	25	1419	1419	<8	2839	14
	39	<8	<8	<8	<8	<8	<8	632	1005	2010	<8	1005	89	710	399	2010	<8	1005	45
	43	<8	10	16	<8	16	16	354	399	710	<8	502	50	502	1005	1265	<8	1127	50

Groups A and B correspond to volunteers immunized with 5 and 10 µg of EV71vac, respectively.

## Discussion

The lack of an efficacious vaccine against EV71 is a major hurdle in preventing the spread of EV71 in Asia-Pacific regions. A report by Cheng et al. [24] revealed EV71vac to be safe and well tolerated that local reactions at the injection-site and adverse systemic effects were mild. In this report, we focus on the effectiveness of EV71vac at eliciting VNA responses against other EV71 genotypes. Although the control group was not stated in the study, as a biosafety monitoring program in NHRI the blood samples collected from >35 R&D staff working in the viral pilot plants were randomly tested every six months for potential exposure to EV71 virus and H5N1 flu virus. The test results so far showed no increase in EV71 neutralizing antibody titers. In addition, during the clinical trial there was no EV71 epidemic occurred in Taiwan (01/2011–03/2012) and each volunteer had filled out the report form during the trials. None of them reported that they had been exposed to EV71 and/or Coxsackie virus infection. Therefore, the increased neutralizing antibody responses are elicited by EV71vac immunization.

The results from current immunogenicity study indicate that a 5-µg dose of EV71vac is highly immunogenic in the tested volunteers. Several important conclusions can therefore be drawn from the study. (1) Based on the rate of seroconversion (≥4-fold increase in neutralizing antibody titers determined by the B4-based virus neutralization assay), >95% of volunteers sero-converted; (2) EV71vac is capable of eliciting strong VNA responses in naive volunteers (those participants who were determined to most likely not have been exposed to or infected with EV71, and four of these five volunteers sero-converted as their IgG titers and Nt increased by more than 4-fold after a single dose of EV71vac); (3) EV71vac induced very strong VNA responses in >85% of volunteers without pre-existing Nt against subgenotype B1, B5 and C4A; (4) EV71vac elicited weak VNA responses (∼20% of participants) against a C4B and CVA16; (5) >90% (51/56) of vaccinated young adults without pre-existing Nt against C2 did not develop VNA responses (Nt<8) against a C2 strain; (6) EV71vac can boost and significantly enhance VNA responses in volunteers who already had pre-vaccination antibodies against EV71 and/or CVA16; and (7) most volunteers vaccinated twice with EV71vac did not show further increase in Nt. To our surprise, a decrease in the GMT of Nt was observed after the booster injection (Tables 1, 2, 3). The increased EV71 neutralizing antibody level after the 1st vaccination may be caused by recall immunity in humans. This indicates the possibility of cross-priming effect. The cross-priming effect is one of benefits from the recall immunity. The immune systems (B- and T-cell) of the host is primed by either exposed to or immunized with target antigen(s) and has generated B- and T-cell immune responses specific to certain epitopes of the target antigen(s). There would be very strong recall immunity from both memory B- and T-cell when the host is exposed to antigen-like molecules containing some of these antigen-specific epitopes.

Clinical trial results from Li et al. [6] and Meng et al. [26] indicated that 1 microgram dose could be sufficient. In our recent review article [23], we reported based on 7 batches of 40 liters pilot-scale production runs, we could produce 50,000 doses of the EV71vac at 1 µg EV71 antigen per dose and the cost was calculated to be 0.4 US dollar/dose. Using our current GMP facility (7,500 sq ft), 1 million doses can be easily produced annually.

Is the current vaccine derived from an EV71 subgenotype B4 strain the right choice? We did not observe poor VNA responses against C2 in EV71vac immunized mice, rabbits and macaques in pre-clinical studies [5], [19], [21]–[23]. These immune responses in animal models were confirmed by other studies [16]–[17]. The C2 strain used in the assay was isolated during the 1998 outbreak and identified as a recombinant virus between EV71 genotype A and Coxsackie virus A8 by Huang *et al.* [12]. The C2 subgenotype virus was not found in Taiwan since 1998, but still circulates in Thailand [4]. Interestingly, Huang *et al.* [12] also suggested that the current B4 vaccine strain and C4B strain were recombinants between EV71 subgenotype B2/B3 and B2/C2, respectively. The occurrence of intra- and inter-serotyptic recombination could cause genetic evolution and antigenic changes that result in decrease virus cross-neutralization activity as observed in the current results.

The weak and poor cross-neutralizing antibody responses against C4B, CVA16 and C2, prompted us to test other EV71 isolates in the laboratories. From the preliminary results, Subjects who were previously found to have Nt<8 against C2 strain were shown to have a >4-fold increase in Nt against another C2 strain (EV71/4643) and another C4B (EV71/1757) strain (Dr. J.R. Wang, unpublished results). To settle these discrepancies, we are currently purifying the virus strains and standardizing their viral titer used in the cross-neutralization assay since we have found that different virus strains could generate different amounts of defective particles that could interfere with the neutralization titer. In our previous studies [5], [9], [20], the ratio of defective to infectious particles can vary from 7∶3 in the current vaccine strain to 1∶2 for CVA16 (5079/TW98).

The current study indicate that preexisting antibody due to stealth infections could interfere the efficacy of the vaccine, so we urgently need a good biomarker to pre-screen naive subjects since most young children in epidemic Asian countries have likely been exposed to and/or infected with EV71 and/or CVA16. The EV71 particle-based specific ELISA may be used as a quick screening test of pre-immune sera from volunteers since at the 1/2000 dilution EV71 negative sera correlates with Nt levels <8.

Although animal immunogenicity study in general is used to evaluate the consistency of immunogenicity among different batches of vaccines, it is of interest to know which animal (rabbit, mouse and macaque) immunogenicity study can be correlated to the potency of EV71vac. Mice immunized with EV71vac mainly produced antibodies against the immunodominant neutralization epitope VP1–43 corresponding to residues 205–225 (FGEHKQEKDLEYGAC) of VP1 [19]–[20]. But our unpublished epitope mapping results show that sera of all participants immunized with EV71vac did not react with the VP1–43 peptide, but recognized conformational neutralization epitopes and an immunodominant linear epitope VP1–49 (residues 241–260). This means that immune responses from mice immunized with EV71vac would not correlate with the potency of the vaccine candidate in human. In contrast, the conformational-dependent neutralizing antibody responses from rabbits and macaques immunized with EV71vac are very similar to those generated in humans. They are characterized by strong virus neutralization titers against subgenotypes B1, B4, B5 and C4A listed in this study, moderate Nt against C4B and C2, but poor responses against CVA16 [5], [17]. Based on the cost of animals and housing requirements, rabbit immunogenicity study could be the choice for an EV71 vaccine potency test. In addition, the immune responses from animal models and human volunteers vaccinated with EV71 vaccine candidate based on B4 subgenotype have indicated that a multi-valent (B4, C2 and CVA16) vaccine against the hand-foot-mouth diseases may be necessary.

Overall, the current results indicate that EV71vac based on an EV71 subgenotype B4 strain is a good vaccine candidate. It is safe, well tolerated and effectively elicits cross-neutralizing antibody responses against EV71 subgenotypes B1, B4, B5 and C4A. We should keep in mind that the target population of the EV71 vaccine is young infant and children younger than 5 years old. The cross-neutralization immune responses to EV71vac in children should be investigated. In addition, the vaccination schedule and the long-term immunogenicity should be assessed as well. Therefore, the current results provide a strong rationale for further evaluations of EV71vac in phase II clinical trials.

## Supporting Information

Protocol S1 **This study is registered with ClinicalTrials.gov with the registration #NCT01268787.** The protocol was approved by Taiwan FDA #9917719 and conducted in compliance with National Taiwan University Hospital (NTUH) IRB# 201007056M and Veteran General Hospital Taipei IRB#201008015MA. The inclusion/exclusion criteria for the subject recruitment and randomization were reported in our recent publication [24].
(PDF)

File S1 **This file describes the Supplementary Materials of Formalin-inactivated EV71 vaccine candidate induced cross-neutralizing antibody against subgenotypes B1, B4, B5 and C4A in adult volunteers.** The contents include: Table S1. Summary of pre- and post-vaccination immune responses from individual subjects. Table S2. Summary of EV71 B genotypes-specific virus neutralizing antibody titers obtained from subjects immunized with the EV71 vaccine. Table S3. Summary of EV71 C4 subgenotypes-specific virus neutralizing antibody titer obtained from human sera immunized with EV71 vaccine. Figure S1. Virus neutralizing antibody responses of young adults immunized with EV71vac against subgenotype B4 (vaccine strain). Figure S1. Virus neutralizing antibody responses of young adults immunized with EV71vac against subgenotype B4 (vaccine strain). Panels A and B show the neutralization titers of sera obtained from volunteers immunized twice with either a 5- or 10-µg dose, respectively. Thirty healthy adults were enrolled in each group. Each spot represents the neutralizing antibody titer for individual sera. The bar corresponds to the geometric mean titer (GMT) of each set of sera.
(DOC)

Checklist S1 **CONSORT checklist of a Phase 1, Randomized, Open-Label Study to Evaluate the Safety and Immunogenicity of Enterovirus 71 Vaccine.**
(DOC)
