# Supplementary material for: Formalin-Inactivated EV71 Vaccine Candidate Induced Cross-Neutralizing Antibody against Subgenotypes B1, B4, B5 and C4A in Adult Volunteers
Source: PLoS One. 2013 Nov 21;8(11):e79783. doi: 10.1371/journal.pone.0079783 (PMC3836818; doi:10.1371/journal.pone.0079783)
Supplement: Protocol S1 — This study is registered with ClinicalTrials.gov with the registration #NCT01268787. The protocol was approved by Taiwan FDA #9917719 and conducted in compliance with National Taiwan University Hospital (NTUH) IRB# 201007056M and Veteran General Hospital Taipei IRB#201008015MA. The inclusion/exclusion criteria for the subject recruitment and randomization were reported in our recent publication [24]. (PDF) [file pone.0079783.s001.pdf]

CLINICAL STUDY PROTOCOL

A Phase I, Randomized, Open-Label Study to Evaluate the Safety and Immunogenicity of EV71 Vaccine Administrated with Adjuvant $AlPO_4$ in Health Volunteers

Protocol No.:	QCR10013
Phase:	I
Study Drug:	EV71 Vaccine
Indication:	Prophylaxis against enterovirus 71 infection
Version:	1.2
Date:	17-Aug-2010

Study Sponsor: National Health Research Institutes
Vaccine Research and Development Center
No 35, Keyan Road, Zhunan Town, Miaoli County 35053, Taiwan (R.O.C.)
TEL: 886-37-246-166
FAX: 886-37-586-401

CONFIDENTIAL

Protocol Synopsis

Ⅰ. Protocol title:

A Phase I, Randomized, Open-Label Study to Evaluate the Safety and Immunogenicity of EV71 Vaccine Administrated with Adjuvant $AlPO_4$ in Health Volunteers

Ⅱ. Objectives:

Enteroviruses (EV) are small RNA (ribonucleic acid) viruses associated with several human and mammalian diseases. It can cause outbreaks of hand-foot-and-mouth disease (HFMD) or herpangina, and is sometimes associated with severe neurologic complications. Several enterovirus epidemics have been reported in Taiwan, and enterovirus 71 (EV-71) infection was associated with most of the serious clinical manifestations.

This study aims to evaluate the safety, reactogenicity and immunogenicity of EV71 vaccine at a 5-μg and 10-μg dose in health volunteers

1. Pimary objective:

- ♦ To evaluate the safety and reactogenicity of the EV71 vaccine at a 5-μg and 10-μg dose.

2. Secondary objective:

(1) To evaluate the immunogenicity in terms of immunoglobulin G (IgG) titers induced by the EV71 vaccine at a 5-μg and 10-μg dose.

(2) To evaluate the immunogenicity in terms of neutralizing antibody titers from baseline induced by the EV71 vaccine at a 5-μg and 10-μg dose.

(3) To identify the immunodominant epitopes of EV71 virus by human serum.

Ⅲ. Test vaccine:

1. Name: EV71 Vaccine

2. Dosage form: Injection, 3 ml sterile suspension/vial (applied for maximum 5 doses at the same time), 20 μg/ml viral protein with adjuvant 600 μg/ml aluminium ($AlPO_4$)

3. Dose:

(1) Group A05: 0.25 ml per dose (5 μg total protein + adjuvant 150 μg $AlPO_4$)

(2) Group B10: 0.5 ml per dose (10 μg total protein + adjuvant 300 μg $AlPO_4$)

4. Dosing schedule: Two intramuscular (i.m.) vaccinations, 21 days apart (on Day 0 and Day 21)

5. Mechanism of action: Preventive vaccination of inactivated EV71 whole virion (EV71vac bulk) to induce antibody against EV71 virus.

6. Pharmacological category: Vaccine

Ⅳ. Developmental phase: phase ■Ⅰ □Ⅱ □Ⅲ(□Ⅲa or □Ⅲb) □Ⅳ □Others

Ⅴ. Study design:

1. ☐Control: ☐placebo

☐active __________

☐other __________

■Uncontrolled

2. Blinding: ■open-label ☐evaluator blind ☐single blind ☐double blind

☐double dummy ☐other ____________

3. Randomized: ■yes ☐no

4. ■Parallel ☐Cross-over ☐Other ____________

5. Duration of treatment: _Two-vaccination regimen, 21 days apart_

6. Titration: ☐forced ☐optional ■none

7. ☐Multi-national ■Multi-center (Taiwan) ☐Single center

Ⅵ. Endpoints

1. Primary endpoint:

- The percentage, intensity and relationship to vaccination of local and systemic signs and symptoms at a 5-μg and 10-μg dose under the following time frames:
  - Solicited adverse events: during 7-day follow-up period
  - Unsolicited adverse events: during 21-day follow-up period
- The occurrence of overall adverse events and serious adverse event at a 5-μg and 10-μg dose during the entire study period.

2. Secondary endpoints:

(1) Observed variables from baseline at a 5-μg and 10-μg dose on Day 21 and 42:
  - Serum IgG titers
  - Serum neutralizing antibody titers
  - Epitope mapping (only on Day 42)

(2) The change in the laboratory results:
  - Hematology
  - Biochemistry

Ⅶ. Selection criteria:

1. Main inclusion criteria:

(1) Female and male ≥ 20 and ≤ 60 years of age at the time of first vaccination.

(2) Subject who is free of obvious health problems as established by medical history and physical examination based on the investigator’s clinical judgment.

(3) Able and willing to comply with the study procedure and give written informed consent.

2. Main exclusion criteria:

(1) Female who is pregnant/lactating or planning to be pregnant, or female of childbearing potential* who is not using medically recognized method of contraception**.

* Other than those who have been surgically sterilized (defined as having undergone hysterectomy or bilateral oophorectomy or bilateral salpingectomy; tubal ligation alone is not considered sufficient) or one year post-menopausal.

** Recognized contraceptive precautions, including intrauterine contraceptive device; oral contraceptives; diaphragm or condom in combination with contraceptive jelly, cream or foam; Norplant® or DepoProvera®, should be conducted until 2 months after completion of the vaccination series.

(2) Subject with body mass index (BMI) > 35.

(3) Subject with fever defined as oral temperature ≥ 37.5˚C at the time of planned vaccination.

(4) Subject with previous known or potential exposure to Enterovirus 71 (EV71).

(5) Subject with a history of herpangina, hand-foot-mouth disease, acute hemorrhagic conjunctivitis or acute gastrointestinal illness associated with enterovirus infection in the past 3 months.

(6) Subject with any confirmed or suspected abnormal immune function, immunosuppressive or immunodeficient condition.

(7) Subject who has been diagnosed with a significant neurological, pulmonary, cardiovascular, hematological, hepatic or renal disorder.

(8) Subject with any abnormal laboratory results at screening.

(9) Subject with a history of hypersensitivity to vaccines, or a history of allergic disease or reactions likely to be exacerbated by any component of the vaccine.

(10) Use of any investigational/non-registered product (including drug, vaccine and invasive medical device) within 30 days prior to vaccination or planned use during the study period.

(11) Administration of any licensed vaccine within 30 days prior to vaccination or planned administration during study period (until 30 days after the second vaccination).

(12) Use of immunoglobulins or any blood products within 3 months prior to vaccination or planned use during the study period (until 30 days after the second vaccination).

(13) Chronic administration (defined as > 14 days) of immunosuppressants or other immunomodulators within 6 months prior to vaccination or planned use during the study period (until 30 days after the second vaccination).

* For corticosteroids, this includes prednisone ≥ 0.5 mg/kg/day or equivalent. Inhaled and topical steroids are allowed.

(14) Chronic or long-term (defined as > 14 days) use of acetylsalicylic acid medication within 6 months prior to vaccination or planned use during the study period (until 30 days after the second vaccination).

(15) Known or suspected with alcohol or substance abuse.

(16) Subject with any medical or psychiatric condition, including the presence of

significant laboratory abnormalities, which is a contraindication to protocol participation based on the judgment of the investigator.

3. Treatment discontinuation:

Withdrawal criteria:

(1) Subject consent withdrawal.

(2) Lost to follow-up.

(3) Administration of prohibited medication/treatment/vaccine.

(4) Any pathological event, clinical adverse event, or any change in the subject's status giving indication to the physicians that further participation in the study may not be the best interest of the subject.

Contraindications to second vaccination:

(1) Subject with any abnormal laboratory results* at safety screening visit (Visit 3).

* Subject with ALT/AST or creatinine > 1 x ULN but < 2.5 x ULN could be re-tested 1 week later.

(2) Pregnancy

(3) Subject with fever defined as oral temperature ≥ 37.5˚C at the time before second vaccination.

(4) Subject with acute disease at the time before the second vaccination (defined as the presence of a moderate or severe illness with or without fever).

Ⅷ. Study procedures:

This is a phase I, prospective, randomized, open-label, two-center study. 60 eligible healthy volunteer will be recruited into this study. After enrollment, all subjects will be randomized to 2 dosing groups, Group A05 or Group B10, in a 1:1 ratio. Both groups will follow the same visit structure and be administrated 2 doses of EV71 vaccine under the dosage below on Day 0 and Day 21, respectively.

- **Group A05:** 0.25 ml per dose (5 µg total protein + adjuvant 150 µg $AlPO_4$)
- **Group B10:** 0.5 ml per dose (10 µg total protein + adjuvant 300 µg $AlPO_4$)

The study recruitment should be carried out in 2 parts, Part I and Part II.

Part I: This part of study will be conducted in one center. It will consist of the first 10 subjects who will have sequential enrollment. Following a blocking factor of safety, these subjects will be recruited in 3 blocks, Block A, Block B and Block C.

- *Block A*: Only the first subject will be assigned to Block A. He/she will be vaccinated with EV 71 vaccine 0.25 ml (5 µg total protein with adjuvant 150 µg $AlPO_4$).
- *Block B*: Block B will be initiated 7 days safety monitoring after the first vaccination of Block A. There will be 2 subjects recruited into this block at the same time, and they will be randomized into two dosing groups, Group A05 and Group B10, respectively.
- *Block C*: Block C will be initiated 7 days safety monitoring after the second vaccination of Block B and 7 subjects will be enrolled into this block at the same time. Three of them will be randomly assigned to Group A05 and the other 4

subjects will be randomly assigned to Group B10.

Part II: This part of study will be conducted in two centers. It will consist of the rest 50 subjects and should be initiated after the DSMB (Data and Safety Monitoring Board) evaluation conducted 14 days after the second vaccination of Block C.

All subjects should complete at least 7 visits, including the last 2 telephone visits. Additional visit, Visit 2a (Day 3) and Visit 4a (Day 24) will be conducted first in Part I for monitoring creatine kinase (CPK) in subjects at the third day after 1st and 2nd vaccination. Whether these 2 visits will be performed again in Part II depended on the DSMB's professional judgments. The immune response, including serum IgG and neutralizing antibody titers, will be assessed at Visit 2, 4 and 5 (baseline, Day 21 and Day 42), and immune epitope mapping of EV71vac will be conducted at Visit 2 and Visit 5. A post-vaccination diary card will be dispensed for the solicited/unsolicited symptoms, and a telephone follow-up will be conducted 7 days after each vaccination and/or 14 days after second vaccination (only for subjects in Block C). The safety of EV71 vaccine will be monitored throughout the whole study period.

The investigator team (Principal Investigator and Co-Investigators) should, on an ongoing basis, review the subject's safety data (including all AE recorded on the diary card and detected during study period) immediately and make medical judgment whether the adverse event is vaccine-related or not. The independent DSMB should monitor all the safety data as well. The second part of study could be conducted only when there is no safety concerns regarding the EV71 vaccine (evaluated on 14 days after the second vaccination of Block C). Moreover, the study recruitment should be suspended if any of the following criteria is met.

(1) Any subject with severe local reaction, including ulceration, abscess or necrosis.

(2) Any subject develops laryngospasm, bronchospasm or anaphylaxis.

(3) Two of more subjects develop severe urticaria.

(4) Any vaccine-related SAE during study period.

All subjects will be asked to preserve the residue blood samples for further research through a separate question in informed consent.

Ⅸ. Concomitant treatment:

1. Permitted:

Any treatment other than those prohibited below may be administrated during this trial. All concomitant treatments except vitamins and dietary supplements should be recorded in the Case Report Form.

2. Prohibited:

(1) The following treatments are prohibited until 30 days after the second vaccination:

- Other vaccine
- Immunoglobulins
- Any blood products
- Immunosuppressants or other immunomodulators*

* For corticosteroids, this includes prednisone $\geq$ 0.5 mg/kg/day or equivalent.

Inhaled and topical steroids are allowed.

- o Chronic or long-term (defined as > 14 days) use of acetylsalicylic acid medication

(2) The following treatments are prohibited during the study period:

- o Investigational/non-registered product (including drug, vaccine and invasive medical device)

Ⅹ. Statistics:

1. Primary hypothesis: ☐superiority ☐non-inferiority ☐equivalence ■other NA

2. Sample size: 60

3. Efficacy population: ☐ITT ☐PP ■other ATP

Safety population: ☐ ITT ☐ PP ■ other Who receives at least one vaccination

4. Statistical method(s) for safety/immunogenicity evaluations:

(1) The major analysis will be performed according to The Total Vaccinated cohort (TVC), According-To-Protocol (ATP) cohort for analysis of immunogenicity and According-To-Protocol (ATP) cohort for analysis of safety.

(2) For descriptive statistics, continuous variables will be presented as mean, standard deviation, median and range; Categorical variables will be presented in frequency table.

(3) All adverse events will be coded through MedDRA system and provided by system of organ and preferred term. The analysis of subjects experienced adverse events/serious adverse events will consist of a standardized tabulation of counts, percentage and 95% confidence interval of all observed adverse events (solicited and unsolicited).

(4) The mean change of immunogenicity parameters include serum IgG and neutralizing antibody titers from baseline will be presented descriptively and assessing by paired T-test or Wilcoxon signed rank test under significance level 0.05. The summary results of immune epitope mapping will be presented descriptively.

(5) The mean change of laboratory parameters from baseline will be presented descriptively and assessing by paired T-test or Wilcoxon signed rank test under significance level 0.05.

5. Planned interim analysis: ■yes ☐no

An interim safety analysis will be conducted 14 days after the second vaccination of Block C. The second part of study could be initiated only after the interim report has been reviewed and approved by DSMB.

XI. Flow Chart

	Screening	1st vaccination	CPK monitoring	Safety screening	2nd vaccination	CPK monitoring	Follow-up		
**Scheduled visit**	1	2	2a^	3	4	4a^	5	6*	7*/ET#
**Day**	-7~0	0 (Baseline)	3±1 (D2~4)	14+3 (D14~17)	21+3 (D21~24)	24+3 (D24~27)	42±3 (D39~45)	126±14 (D112~140)	210±14 (D196~224)
Informed consent	X								
Inclusion/Exclusion criteria	X	X†							
Withdrawal criteria		X†		X	X†		X	X*	
Contraindication for second vaccination				X	X†				
Randomization		X†							
Demographics	X								
Medical history	X								
Physical examination[1]	X[1]	X†		X	X†		X		
Oral temperature	X	X†		X	X†		X		
Urine pregnancy test[2]	X	X†			X				
Lab (Safety)									
Hematology[3]	X			X			X		
Biochemistry[4]	X		X^	X		X^	X		
Lab (Immunogenicity)									
IgG titers		X†			X†		X		
Neutralizing antibody titers		X†			X†		X		
Epitope mapping		X†					X		
Vaccination									
Administration		X			X				
2-hour observation		X			X				
7-day phone contact		X*			X**				
Diary card									
Distribution		X			X				
Return					X		X		
Concomitant medications	X	X		X	X		X	X*	X*
Adverse events		X		X	X		X	X*	X*
Study completion									X*

[1] Body height will be only measured at Visit 1.

[2] Pregnancy test will be only conducted in female subject of childbearing potential.

[3] Hematology tests include CBC, such as Hb, Hct, RBC count, WBC count, WBC differential and platelet count.

[4] Biochemistry tests include ALT, AST, γGT, alkaline phosphatase, total bilirubin, total protein, albumin, BUN, creatinine, uric acid, total cholesterol, triglyceride and glucose (fasting).

† These procedures should be conducted before vaccination.

* These study procedures will be conducted through telephone contact. ** For subjects in Block C, a 14-day telephone follow-up will also be taken place. ^ Creatine kinase (CPK) assay at Visit 2a and Visit 4a in Part I should be monitored at the 3rd days after each vaccination, the results should be evaluated by DSMB to decide if CPK should be monitored in Part II.

ET: early termination (The final telephone follow-up may be omitted if a subject is withdrawn as a reason other than safety.)

Table of Content

PROTOCOL SYNOPSIS **2**
TABLE OF CONTENT **9**
LIST OF FIGURE AND TABLES **11**
LIST OF ABBREVIATIONS AND TERMS **12**
1. INTRODUCTION **14**
1.1. BACKGROUND 14
1.2. STUDY RATIONALE 14
1.3. PRECLINICAL STUDY 15
2. STUDY OBJECTIVE **16**
2.1. PRIMARY OBJECTIVE 16
2.2. SECONDARY OBJECTIVE 16
3. STUDY DESIGN **16**
3.1. STUDY SUBJECT ENROLLMENT PROCESS: 16
3.2. STUDY ARRANGEMENT: 18
3.3. INTERIM ANALYSIS: 18
3.4. ANCILLARY STUDY 18
4. SUBJECT ELIGIBILITY **18**
4.1. NUMBER AND SOURCE 18
4.2. MAIN INCLUSION/EXCLUSION CRITERIA: 18
4.2.1. Main inclusion criteria: *18*
4.2.2. Main exclusion criteria: *19*
4.3. CRITERIA FOR TREATMENT DISCONTINUATION OR REMOVAL FROM THE STUDY 20
4.3.1. Withdrawal criteria *20*
4.3.2. Contraindications to second vaccination *20*
5. STUDY PROCEDURE **21**
5.1. VISIT 1 (DAY -7~0) – SCREENING 23
5.2. VISIT 2 (DAY 0) – 1ST VACCINATION 24
5.3. VISIT 3 (DAY 14+3) – SAFETY SCREENING 25
5.4. VISIT 4 (DAY 21+3) – 2ND VACCINATION 25
5.5. VISIT 5~7 – FOLLOW-UP 26
5.5.1. Visit 5 (Day 42±3) *26*
5.5.2. Visit 6 and 7 (Day 126±14 and 210±14) – Telephone follow-up *27*
5.6. EARLY TERMINATION (ET) 27
6. STUDY DRUG **27**
6.1. TREATMENT ASSIGNMENT 27
6.2. TREATMENT BLINDING 28
6.3. DESCRIPTION OF STUDY VACCINE 28
6.4. DOSAGE AND ADMINISTRATION OF STUDY VACCINE 29
6.5. CONCOMITANT TREATMENT 30
6.5.1. Permitted treatment *30*
6.5.2. Prohibited treatment *30*
6.6. RESCUE MEDICATION 31
6.7. DRUG ACCOUNTABILITY 31
6.8. TREATMENT EXPOSURE AND COMPLIANCE 31

7. ASSESSMENT PARAMETERS 32
7.1. DEMOGRAPHICS / OTHER BASELINE CHARACTERISTICS 32
7.2. IMMUNOGENICITY 32
7.2.1. EV71 specific IgG antibody titer 32
7.2.2. Neutralizing antibody titres 32
7.2.3. Epitope mapping 33
7.3. SAFETY 33
7.3.1. Physical examination / body temperature 33
7.3.2. Urine pregnancy test 33
7.3.3. Selected laboratory safety tests 34
7.3.4. Self-evaluation (solicited/unsolicited symptoms) 34
7.3.5. Adverse events (AEs) 35
7.3.6. Serious adverse events (SAEs) 39
7.4. SERA CRYOPRESERVATION 39
8. SAFETY MONITORING 39
8.1. SERIOUS ADVERSE EVENTS REPORTING REQUIREMENT AND EMERGENCY PROCEDURES 39
8.2. PREGNANCIES 40
8.3. DATA SAFETY MONITORING BOARD (DSMB) 40
8.3.1. DSMB responsibilities 40
8.3.2. DSMB process 41
8.3.3. DSMB meeting format 41
8.3.4. Meeting materials 41
8.3.5. Reports from the DSMB 42
8.4. STUDY STOPPING CRITERIA 42
9. STUDY ENDPOINTS 42
9.1. PRIMARY ENDPOINT 42
9.2. SECONDARY ENDPOINTS 42
10. STATISTICS 43
10.1. STUDY COHORTS TO BE EVALUATED 43
10.1.1. Total Vaccinated cohort (TVC) 43
10.1.2. According-To-Protocol (ATP) cohort for analysis of immunogenicity 43
10.1.3. According-To-Protocol (ATP) cohort for analysis of safety 43
10.2. BASELINE AND DEMOGRAPHIC CHARACTERISTICS 43
10.3. ANALYSIS OF THE PRIMARY OBJECTIVES 43
10.3.1. Variables 44
10.3.2. Statistical hypothesis, model, and method of analysis 44
10.4. ANALYSIS OF SECONDARY OBJECTIVES 44
10.4.1. Variables 44
10.4.2. Safety 45
10.5. HANDLING OF MISSING DATA 45
10.6. SAMPLE SIZE AND POWER CONSIDERATIONS 45
10.7. PLANNED INTERIM ANALYSIS 45
11. COMPLIANCE OF ETHICS 46
11.1. ETHICAL CONDUCT OF THE STUDY 46
11.2. INSTITUTIONAL REVIEW BOARD (IRB)/ETHICS COMMITTEE (EC) 46
11.3. INFORMED CONSENT PROCESS AND SUBJECT INFORMATION 46
12. INSURANCE AND INDEMNITY 46
13. SOURCES DOCUMENTS 47
14. CASE REPORT FORM 47
15. CONTROL AND ASSURANCE OF STUDY QUALITY 47

15.1. ASSIGNMENT OF SITE MONITOR....47
15.2. DATA QUALITY ASSURANCE....47
16. PROTOCOL DEVIATIONS....48
17. PROTOCOL AMENDMENTS....48
18. TERMINATION OF STUDY....48
19. RETENTION OF RECORDS AND CONFIDENTIALITY....48
20. USE OF INFORMATION AND PUBLICATION....49
21. REFERENCES....49
APPENDIX I IMMUNOGENICITY SAMPLING GUIDELINE (CENTRAL LABORATORY)....50
APPENDIX II....51

List of figure and tables

FIGURE 3-1	STUDY SUBJECT ENROLLMENT PROCESS:	17
TABLE 5-1	STUDY PROCEDURES:	22
TABLE 5-2	INTERVALS BETWEEN STUDY VISITS:	23
TABLE 6-1	NUMBER OF PARTICIPANTS ASSIGNED TO EACH BLOCK:	28
TABLE 6-2	DOSAGE AND ADMINISTRATION OF STUDY VACCINE:	30
TABLE 7-1	SOLICITED LOCAL AND GENERAL SYMPTOMS /ADVERSE EVENTS:	35
TABLE 7-2	INTENSITY SCALES OF SOLICITED/UNSOLICITED SYMPTOMS:	37
TABLE 7-3	INTENSITY SCALES OF AE:	38
TABLE 7-4	THE RELATIONSHIP BETWEEN AE AND STUDY VACCINE:	38

List of Abbreviations and Terms

AE(s)	Adverse Event(s)
Al	Aluminium
$AlPO_4$	Aluminium Phosphate
ALT	Alanine Aminotransferase
AST	Aspartate Transaminase
BDM	Biostatistics & Data Management
BMI	Body Mass Index
BUN	Blood Urea Nitrogen
CA16	Coxsackievirus A16
CBC	Complete Blood Count
DOH	Department of Health
CRC	Clinical Research Coordinator
CRF	Case Report Form
CRO	Contract Research Organization
DBP	Diastolic Blood Pressure
DSMB	Data and Safety Monitoring Board
EC	Ethics Committee
ELISA	Enzyme-Linked Immunosorbent Assay
ET	Early Termination
EV	Enteroviruses
γ GT	γ Glutamyl Transferase
GCP	Good Clinical Practice
GMT	Neutralization Geometric Mean
Hct	Hematocrit
Hb	Hemoglobin
HBL	Hayashibara Biochemical Laboratories
HFMD	Hand-Foot-and-Mouth Disease
HRP	Horseradish Peroxidase
IB	Investigational Brochure
ICF	Informed Consent Form
ICH	International Conference on Harmonization
IgG	Immunoglobulin G
i.m.	Intramuscular

IRB	Institutional Review Board
NA	Not Applicable
NHRI	National Health Research Institutes
PBS	Phosphate Buffer Saline
PI	Principal Investigator
RBC	Red Blood Cell
RNA	Ribonucleic Acid
RT	Room Temperature
SAE(s)	Serious Adverse Event(s)
SBP	Systolic Blood Pressure
SD	Standard Deviation
SOP	Standard Operating Procedure
Taiwan CDC	Taiwan Center of Disease Control
TFDA	Taiwan Food and Drug Administration
ULN	Upper Limit of The Normal
VRDC	Vaccine Research and Development Center
WBC	White Blood Cell

1. Introduction

1.1. Background

The genus *Enterovirus* belongs to a group of small RNA virus and is often found in gastrointestinal and respiratory tract. People can be infected by direct contact with the secretions of an infected person or a contaminated surface or objects. Generally speaking, enterovirus infection is considered slight and self-limiting disease; however, the subtype 71 is the exception.

Since Enterovirus 71 (EV71) was first recognized in California in 1969, world–wide reports have followed. In the last decade of 20th century, trend of EV71 outbreak was mainly observed in the Asia-Pacific regions.[1] In 1998, EV 71 caused an unprecedented outbreak in Taiwan and the following smaller outbreaks recurred in the following ten years as seasonal epidemic. People infected with EV 71 were recognized because of clinical manifestations of hand, foot, and mouth disease (HFMD) and the rapid deaths of children fewer than five years old. HFMD caused by many human enteroviruses, including coxsackieviruses A4, A5, A8, A10, A16, B3, and B7 and enterovirus 71. Of these, human EV71 and coxsackievirus A16 (CA16) are two major causative agents of HFMD. EV71 infection is more frequently associated with serious neurological complications and fatalities.[2-3] HFMD is a common rash illness in children and infants and it sometimes cause severe complications such as encephalitis and poliomyelitis-like paralysis, mainly in young children and infants. Among these patients, more than 320 children were hospitalized with suspected meningitis, encephalitis, or acute flaccid paralysis, and at least 55 died, suggesting neurovirulence of the pathogen.[4] Virologic and pathologic studies indicated that EV71 was the most important agent related to severe and fatal cases and that a neurogenic complication was involved in the pathogenesis of cardiopulmonary collapse resulting from fulminate EV71 infection.[5-6]

The chief symptom of neurologic complication caused by EV71 including in 1998 Taiwan outbreak was rhombencephalitis, which had a fatality rate of 14 percent. The most common initial symptoms were myoclonic jerks, and MRI usually showed evidence of brainstem involvement which was also found in autopsy reports of Malaysian patients with EV71 infection. In contrast to the earlier epidemics of EV 71 infections, the Malaysian and Taiwanese epidemics were characterized by rhombencephalitis. This change may indicate the reemergence of virulent strains of EV 71 with serious neurologic effects or the emergence of a new strain.[7] Outbreak of Taiwan makes the world pay more attention to such a fatal epidemic for young children. And many groups in Taiwan are studying the molecular genetics of strains involved in the epidemics.

1.2. Study rationale

Currently there are no vaccines or effective anti-viral drugs for EV71 treatment or prophylaxis, only supportive care can be conducted in patients acquired with EV71. According to seroepidemiologic study, more than half of children in Taiwan have no resistance to EV71, it suggested that in the future large EV71 outbreaks for the second time in Taiwan is still a possibility. If there is no effective way to control EV71 distribution, this will make the whole society in a panic and stress condition, especially in families with small children. For this reason, development of new antiviral drugs or vaccine for EV71 is high priority for high prevalence countries or regions, and vaccination of children under 5

years of age may be warranted.[5]

Taiwan Center of Disease Control (Taiwan CDC) has made significant research progress in the bioprocess for producing cell-cultured-based whole-virion EV71 candidate vaccine using the roller-bottle technology. To carry-on the development of a vaccine against EV71 infection, Vaccine Research and Development Center (VRDC) at National Health Research Institute has the basic technology through technology transfer agreement with Taiwan CDC. VRDC also initiates the project of Taiwan Human Vaccine R&D Programs funded by Department of Health (DOH).

After obtaining the virus strain and Vero cells from Taiwan CDC, VRDC has established working cell and working virus bank for ensuring the adequate supply of equivalent quality of starting materials. The preparation and production processes were conducted in cGMP facility of VRDC and serum-free production process was first adopted by VRDC for generating higher quality and safety of EV71 vaccine. As so far, preclinical study has been completed and the preliminary data showed that EV71vac is safe enough to elicit functional antibody in animal model.

1.3. Preclinical study

Toxicity study:

To predict major organs of toxicity in humans, toxicity studies in both rodent (rats) and non-rodent (rabbits) animal models were performed. No matter in single high-dose (20 μg EV71vec/ 600 μg alum) acute toxicity study in rat model or repeated dose toxicity study in rat and rabbit model, vaccine-related toxicity or any clinical abnormality sign were not observed.

Immunogenicity studies:

Potency of inactivated E59 whole virion (bulk of EV71vac) was evaluated in mouse immunogenicity study, the EV71-specific IgG titer and the virus neutralization titer were determined. After only 0.2 μg of total protein or two doses with higher dosage of immunization, the specific IgG antibody titer generated in mice could attain 10^3 or even more. The results shows that bulk of EV71vac could induce strong immune responses.

For further assessing the immune responses elicited by inactivated EV71 virion, VRDC used rats (rodent), rabbits (non-rodent) as models to evaluate the immunogenicity of vaccine formulated with adjuvant such as aluminum phosphate ($AlPO_4$).

In rat immunogenicity studies, the EV71 vaccine could elicit anti-EV71 antibody response in rats. By two or three doses of immunization with low or high dosage (10/300 or 20/600; μg of EV71/μg of alum), the specific IgG antibody titer and virus neutralization titer generated in rats could achieve over 10^4 and 10^2, respectively. The virus neutralization geometric mean (GMT) titers were found to be greater than 100 and specific IgG titer was also observed to be over 10^3. The same study procedure was performed in rabbit immunogenicity studies. The specific IgG antibody titer and virus neutralization titer generated in rats could achieve over 10^5 and 10^3, respectively. Therefore, EV71vac can elicit functional antibody and is immunogenic in both rat and rabbit model.

Further detail about preclinical studies is described in Investigational Brochure (IB).

2. Study Objective

Enteroviruses (EV) are small RNA viruses associated with several human and mammalian diseases. It can cause outbreaks of hand-foot-and-mouth disease (HFMD) or herpangina, and is sometimes associated with severe neurologic complications. Although several enterovirus epidemics have been reported in Taiwan, enterovirus 71 (EV-71) infection was associated with most of the serious clinical manifestations.

This study aims to evaluate the safety, reactogenicity and immunogenicity of EV71 vaccine at a 5-μg and10-μg dose in health volunteers.

2.1. Primary objective

- To evaluate the safety and reactogenicity of the EV71 vaccine at a 5-μg and 10-μg dose.

2.2. Secondary objective

(1) To evaluate the immunogenicity in terms of IgG titers induced by the EV71 vaccine at a 5-μg and 10-μg dose.

(2) To evaluate the immunogenicity in terms of neutralizing antibody titers from baseline induced by the EV71 vaccine at a 5-μg and 10-μg dose.

(3) To identify the immunodominant epitopes of EV71 virus by human serum.

3. Study Design

3.1. Study subject enrollment process:

This is a phase I, prospective, randomized, open-label, two-center study. 60 eligible healthy volunteer will be recruited into this study. After enrollment, all subjects will be randomized to 2 dosing groups, Group A05 or Group B10, in a 1:1 ratio. Both groups will follow the same visit structure and be administrated 2 doses of EV71 vaccine under the dosage below on Day 0 and Day 21, respectively (refer Section 6.3).

- **Group A05:** 0.25 ml per dose (5 μg total protein + adjuvant 150 μg $AlPO_4$)
- **Group B10:** 0.5 ml per dose (10 μg total protein + adjuvant 300 μg $AlPO_4$)

All subjects will be observed for 2 hours after each vaccination and a telephone contact will be conducted for safety data 7 days and/or 14 days after that.

Figure 3-1 **Study subject enrollment process:**

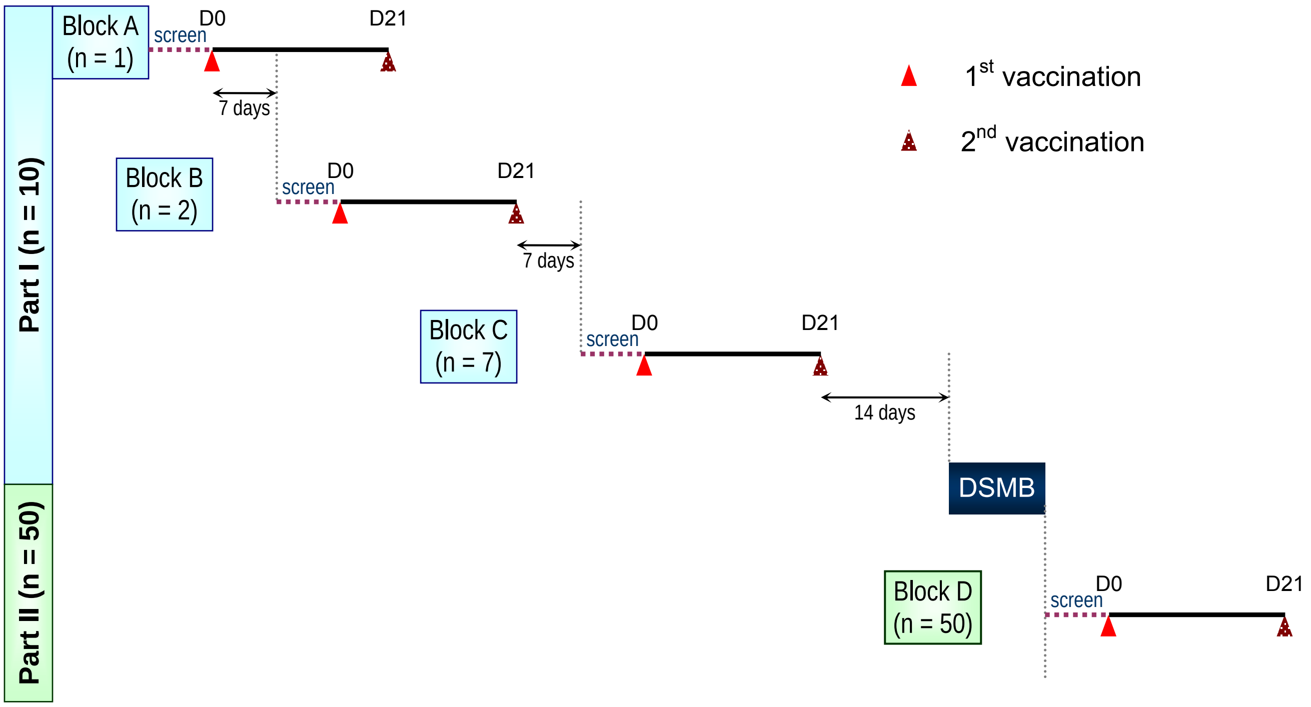

The study recruitment should be carried out in 2 parts, Part I and Part II.

Part I: This part of study will be conducted in one center. It will consist of the first 10 subjects who will have sequential enrollment. Following a blocking factor of safety, these subjects will be recruited in 3 blocks, Block A, Block B and Block C.

- *Block A*: Only the first subject will be assigned to Block A. He/she will be vaccinated with EV 71 vaccine 0.25 ml (5 μg total protein with adjuvant 150 μg $AlPO_4$).
- *Block B*: Block B will be initiated 7 days safety monitoring after the first vaccination of Block A. There will be 2 subjects recruited into this block at the same time, and they will be randomized into two dosing groups, Group A05 and Group B10, respectively.
- *Block C*: Block C will be initiated 7 days safety monitoring after the second vaccination of Block B and 7 subjects will be enrolled into this block at the same time. Three of them will be randomly assigned to Group A05 and the other 4 subjects will be randomly assigned to Group B10.

The investigator team (Principal Investigator and Co-Investigators) should, on an ongoing basis, review the subject's safety data, including all solicited/unsolicited symptoms recorded on the diary card and all AE detected during study period, immediately and make medical judgment whether the adverse event is vaccine-related or not. Block B and Block C will only be taken place after the investigator confirms the initial safety of prior block.

Part II: This part of study will be conducted in two centers. It will consist of the rest 50 subjects and should be initiated after the DSMB (Data and Safety Monitoring Board) evaluation conducted 14 days after the second vaccination of Block C. Subjects in this part will be randomized equally to Group A05 and Group B10.

3.2. Study arrangement:

The study duration of each participant is approximately 210 days. All of them should complete 7 visits, including the last 2 telephone visits. The vaccine will be administrated at Visit 2 and Visit 4 (Day 0 and Day 21). The immune response, including serum IgG and neutralizing antibody titers, will be assessed at Visit 2, 4 and 5 (baseline, Day 21 and Day 42), and immune epitope mapping of EV71vac will be conducted at Visit 2 and Visit 5. A post-vaccination diary card will be dispensed for the solicited/unsolicited symptoms, and a telephone follow-up will be conducted 7 days after each vaccination and/or 14 days after second vaccination (only for subjects in Block C). The safety of EV71 vaccine will be monitored throughout the whole study period.

3.3. Interim analysis:

An interim safety analysis will be conducted 14 days after the second vaccination of Block C. The independent DSMB should monitor all the safety data for this study, including the interim safety data. The second part of study could be initiated only when there is no safety concerns regarding the EV71 vaccine (evaluated on 14 days after the second vaccination of Block C). Moreover, the study may be discontinued or suspended anytime due to any safety concern (refer Section 8.3).

3.4. Ancillary study

All subjects will be asked to preserve the residue blood samples for further research through a separate question in the informed consent in accordance with local ethical and regulatory requirements. The samples will be processed and stored in a -80°C freezer only after agreement. The further research will be able to conduct after getting approval from IRB/EC and health/regulatory authorities.

4. Subject Eligibility

4.1. Number and source

This study will be conducted in 60 healthy volunteers. There will be two study centers in Taiwan and the study participants could be recruited through advertising.

4.2. Main inclusion/exclusion criteria:

The inclusion/exclusion criteria should be checked at the time of study entry.

4.2.1. Main inclusion criteria:

Subjects must meet all of the following criteria:

(1) Female and male ≥ 20 and ≤ 60 years of age at the time of first vaccination.

(2) Subject who is free of obvious health problems as established by medical history and

physical examination based on the investigator's clinical judgment.

(3) Able and willing to comply with the study procedure and give written informed consent.

4.2.2. Main exclusion criteria:

Subjects will be excluded from the study for any of the following reasons:

(1) Female who is pregnant/lactating or planning to be pregnant, or female of childbearing potential* who is not using medically recognized method of contraception**.

* Other than those who have been surgically sterilized (defined as having undergone hysterectomy or bilateral oophorectomy or bilateral salpingectomy; tubal ligation alone is not considered sufficient) or one year post-menopausal.

** Recognized contraceptive precautions, including intrauterine contraceptive device; oral contraceptives; diaphragm or condom in combination with contraceptive jelly, cream or foam; Norplant® or DepoProvera®, should be conducted until 2 months after completion of the vaccination series.

(2) Subject with body mass index (BMI) > 35.

(3) Subject with fever defined as oral temperature ≥ 37.5˚C at the time of planned vaccination.

(4) Subject with previous known or potential exposure to Enterovirus 71 (EV71).

(5) Subject with a history of herpangina, hand-foot-mouth disease, acute hemorrhagic conjunctivitis or acute gastrointestinal illness associated with enterovirus infection in the past 3 months.

(6) Subject with any confirmed or suspected abnormal immune function, immunosuppressive or immunodeficient condition.

(7) Subject who has been diagnosed with a significant neurological, pulmonary, cardiovascular, hematological, hepatic or renal disorder.

(8) Subject with any abnormal laboratory results at screening.

(9) Subject with a history of hypersensitivity to vaccines, or a history of allergic disease or reactions likely to be exacerbated by any component of the vaccine.

(10) Use of any investigational/non-registered product (including drug, vaccine and invasive medical device) within 30 days prior to vaccination or planned use during the study period.

(11) Administration of any licensed vaccine within 30 days prior to vaccination or planned administration during study period (until 30 days after the second vaccination).

(12) Use of immunoglobulins or any blood products within 3 months prior to vaccination or planned use during the study period (until 30 days after the second vaccination).

(13) Chronic administration (defined as > 14 days) of immunosuppressants or other immunomodulators within 6 months prior to vaccination or planned use during the study period (until 30 days after the second vaccination).

* For corticosteroids, this includes prednisone ≥ 0.5 mg/kg/day or equivalent. Inhaled and topical steroids are allowed.

(14) Chronic or long-term (defined as > 14 days) use of acetylsalicylic acid medication within 6 months prior to vaccination or planned use during the study period (until 30 days after the second vaccination).

(15) Known or suspected with alcohol or substance abuse.

(16) Subject with any medical or psychiatric condition, including the presence of significant laboratory abnormalities, which is a contraindication to protocol participation based on the judgment of the investigator.

4.3. Criteria for treatment discontinuation or removal from the study

4.3.1. Withdrawal criteria

Discontinuation from study is defined as discontinuation of all study visit and examinations. Participants may voluntarily withdraw from the study for any reason at any time. They may be considered withdrawn if they state an intention to withdraw, or fail to return for visits, or become lost to follow up for any other reason. Generally, subjects must be withdrawn from the study for any of the following reasons:

(1) Subject consent withdrawal.

(2) Lost to follow-up

(3) Administration of prohibited medication/treatment/vaccine listed in Section 6.5.1.

(4) Any pathological event, clinical adverse event, or any change in the subject's status giving indication to the physicians that further participation in the study may not be the best interest of the subject.

These criteria should be checked at each visit subsequent to the first visit and before any study related activity has been conducted. All subjects enrolled will be followed till Day 210. Protocol violations should not lead to subject withdrawal unless they indicate a significant risk to the subject's safety.

If any subject discontinues the study prematurely, the investigator must determine the primary reason for a subject's premature withdrawal and record this information in the medical chart and Off Study Form. For those who are withdrawn due to adverse event(s) or serious adverse event, the event should be followed until resolution or the event is considered stable.

A final safety follow-up conducted through telephone call(s) should be arranged for these subjects as soon as possible. However, for a subject withdrawn within the regular study visit as a reason other than AE, the telephone follow-up could be omitted and no further information has to be collected since last visit (see Section 5.6). Moreover, for those who are lost to follow-up, investigators should make every effort to contact the subjects and document the steps taken, such as dates of telephone calls, in the source documents.

Subjects who are prematurely withdrawn from the study will not be replaced by an equal number of newly enrolled subjects.

4.3.2. Contraindications to second vaccination

The following conditions constitute a contraindication to vaccination and those should be

checked prior to second vaccination (at Visit 3 and Visit 4):

(1) Subject with any abnormal laboratory results* at safety screening visit (Visit 3):

* Subject with ALT/AST or creatinine > 1 x ULN but < 2.5 x ULN could be re-tested 1 week later.

(2) Pregnancy

(3) Subject with fever defined as oral temperature ≥ 37.5˚C at the time before second vaccination.

(4) Subject with acute disease at the time before the second vaccination (defined as the presence of a moderate or severe illness** with or without fever).

** All vaccines can be administered to persons with a minor illness such as diarrhea, mild upper respiratory infection with low-grade febrile illness, i.e. oral temperature < 37.5°C.

Once a subject meets any of the contraindication, the second vaccination can be administrated at the later day, within a 3-day delay (Day 21 to Day 24), or be withdrawn at investigator's discretion. The investigator should document on the Vaccination page of the CRF, the possible reasons for delay administration/withdrawal (AE, SAE or other) and whether the decision to discontinue further vaccination was made by the subject or the investigator.

Any subject who receive the vaccination with dosage deviation or who does not receive the second vaccination on schedule may not necessarily be withdrawn from the study as further study procedures and the follow-up visits may be performed.

5. Study Procedure

This is a phase I, prospective, randomized, open-label, two-center study. A total 60 healthy volunteers will be screened for baseline characteristic inclusion/exclusion criteria after providing a written informed consent. Eligible subjects will be recruited and equally randomized to either receive 2 doses of EV71 vaccine 0.25 ml or 0.5 ml. All subjects should be followed till Day 210. Assessment schedule shown in Table 5-1 lists all study procedures that should be conducted for safety and immunogenicity assessments and the signs "X" indicate when they must be taken place.

Table 5-1 Study procedures:

Below is a list of all study procedures through the study period and the signs "X" indicate when the procedures are performed.

	Screening	1st vaccination	CPK monitoring	Safety screening	2nd vaccination	CPK monitoring	Follow-up		
**Scheduled visit**	1	2	2a^	3	4	4a^	5	6*	7*/ET#
**Day**	-7~0	0 (Baseline)	3±1 (D2~4)	14+3 (D14~17)	21+3 (D21~24)	24+3 (D24~27)	42±3 (D39~45)	126±14 (D112~140)	210±14 (D196~224)
Informed consent	X								
Inclusion/Exclusion criteria	X	X†							
Withdrawal criteria		X†		X	X†		X	X*	
Contraindication for second vaccination				X	X†				
Randomization		X†							
Demographics	X								
Medical history	X								
Physical examination[1]	X[1]	X†		X	X†		X		
Oral temperature	X	X†		X	X†		X		
Urine pregnancy test[2]	X	X†			X				
Lab (Safety)									
Hematology[3]	X			X			X		
Biochemistry[4]	X		X^	X		X^	X		
Lab (Immunogenicity)									
IgG titers		X†			X†		X		
Neutralizing antibody titers		X†			X†		X		
Epitope mapping		X†					X		
Vaccination									
Administration		X			X				
2-hour observation		X			X				
7-day phone contact		X*			X**				
Diary card									
Distribution		X			X				
Return					X		X		
Concomitant medications	X	X		X	X		X	X*	X*
Adverse events		X		X	X		X	X*	X*
Study completion									X*

[1] Body height will be only measured at Visit 1.

[2] Pregnancy test will be only conducted in female subject of childbearing potential.

[3] Hematology tests include CBC, such as Hb, Hct, RBC count, WBC count, WBC differential and platelet count.

[4] Biochemistry tests include ALT, AST, γGT, alkaline phosphatase, total bilirubin, total protein, albumin, BUN, creatinine, uric acid, total cholesterol, triglyceride and glucose (fasting).

† These procedures should be conducted before vaccination.

* These study procedures will be conducted through telephone contact. ** For subjects in Block C, a 14-day telephone follow-up will also be taken place. ^ Creatine kinase (CPK) assay at Visit 2a and Visit 4a in Part I should be monitored at the 3rd days after each vaccination, the results should be evaluated by DSMB to decide if CPK should be monitored in Part II.

ET: early termination (The final telephone follow-up may be omitted if a subject is withdrawn as a reason other than safety.)

All data obtained from those assessments must be supported in the subject's source documentation and entered into the database from separate source documents. For the immunogenicity assessments, the data will be entered through the reports from central laboratory directly.

During the study period, subjects should be seen for all visits on the designated days or within the time window. Table 5-2 shows the Intervals between study visits. It is the investigator's responsibility to ensure that the intervals between visits/contacts are strictly followed.

Table 5-2 Intervals between study visits:

	Interval	Length of interval
1	(Visit 1 → Visit 2)	Within 7 days
2	(Visit 2 → Visit 2a)	3 ± 1 days
3	(Visit 2 → Visit 3)	14 + 3 days
4	(Visit 2 → Visit 4)	21 + 3 days
5	(Visit 2 → Visit 4a)	24 + 3 days
6	(Visit 2 → Visit 5)	42 ± 3 days
7	(Visit 2 → Visit 6)	126 ± 14 days
8	(Visit 2 → Visit 7)	210 ± 14 days

5.1. Visit 1 (Day -7~0) – Screening

At Visit 1, informed consent must be obtained prior to performing any procedures related to the study and after the healthy volunteer has received sufficient information about the study, the opportunity to ask any questions, and consider the options.

Subjects who have been screened should be listed on the Screening Log. Moreover, all screening procedures conducted and the primary reason for not continuing must be documented in the CRF, but no data will be entered into the database for all screened subjects who do not meet criteria for entering the study. No CRF other than the screening part should be completed for these subjects. The following study procedures will be conducted for screening assessments.

(1) Record date of informed consent signed. Document in the subject's medical chart that he/she is participating in this study, informed consent has been obtained and a copy of the consent has been given to the subject.

(2) Check inclusion/exclusion criteria.

(3) Collect the demographic data, including date of birth, age, gender and ethnicity.

(4) Collect relevant medical history and concurrent diseases. Where possible, diagnoses and not symptoms should be recorded.

(5) Conduct a complete physical examination including the measurement of weight and height. Measure and record the oral temperature.

(6) Collect urine samples for pregnancy test (in females of childbearing potential only).

(7) Collect approximately 8 ml of whole blood sample for following local laboratory tests.

- Hematological tests: CBC, including Hb, Hct, RBC count, WBC count, WBC differential and platelet count.
- Biochemistry tests: ALT, AST, γGT, alkaline phosphatase, total bilirubin, total protein, albumin, BUN, creatinine, uric acid, total cholesterol, triglyceride and glucose (fasting).

(8) Review prior and concomitant medications. All medications, including vaccine(s), taken during the 30-day period before vaccination should be recorded.

5.2. Visit 2 (Day 0) – 1st vaccination

The first dose of study vaccine should be administrated within 1 week after the screening visit. All subjects should complete the following study procedures **prior to vaccination**.

(1) Check inclusion/exclusion criteria.

(2) Check withdrawal criteria.

(3) Conduct randomization of the qualified participant into one of the following dosing groups.

- **Group A05:** 0.25 ml per dose (5 μg total protein + adjuvant 150 μg $AlPO_4$)
- **Group B10:** 0.5 ml per dose (10 μg total protein + adjuvant 300 μg $AlPO_4$)

(4) Conduct a complete physical examination with an exception of body height. Measure and record the oral temperature.

(5) Collect urine samples for pregnancy test (in females of childbearing potential only).

(6) Collect approximately 7 ml of whole blood sample for following central laboratory tests.

- Immunogenicity tests: IgG and neutralizing antibody titers, and epitope mapping.

The study vaccine will be administrated intramuscularly (i.m.) in the deltoid of the non-dominant arm. **After vaccination**:

(1) Observe closely during vaccination and at least 2 hours after vaccine administration. An appropriate medical treatment should be readily available in case of a rare anaphylactic reaction following vaccination.

(2) Conduct a telephone follow-up 7 days after vaccine administration.

(3) Dispense thermometer and self-evaluation diary card. Instruct the subjects to monitor body temperature and complete the diary correctly.
Any solicited symptoms and local and/or general adverse events occurring during a 7 days post-vaccination period, and any unsolicited symptoms occurring during a 21 days post-vaccination period will be recorded on the diary card. The subjects will be instructed to contact the investigator or their delegates immediately if symptoms should manifest or in case of any signs or symptoms occurring which the subject perceives as serious.

(4) Record concomitant medication(s)/vaccine(s) taken since the previous visit and during vaccination period.

(5) Record and report adverse or serious adverse event if any one has occurred since the previous visit.

5.3. Visit 2a (Day 3± 1) – CPK Monitoring

Creatine kinase (CPK) monitoring will be performed in 10 subjects in Part I for 1st vaccination on Day 3 with an allowed a time window of 1 day. Whole blood sample for CPK test will be collected and analyzed by local laboratory.

5.4. Visit 3 (Day 14+3) – Safety screening

A safety screening will be conducted for second vaccination on Day 14 with an allowed delay of 3 days. The following study procedures should be performed in this visit.

(1) Check withdrawal criteria.

(2) Check contraindications to second vaccination.

(3) Conduct a complete physical examination with an exception of body height. Measure and record the oral temperature.

(4) Collect approximately 8 ml of whole blood sample for following local laboratory tests.
- o Hematological tests: the same as screening visit
- o Biochemistry tests: the same as screening visit

* Re-test may be conducted 1 week later (see Section 4.3.2).

(5) Record concomitant medication(s)/vaccine(s) taken since the previous visit.

(6) Record and report adverse or serious adverse event if any one has occurred since the previous visit.

5.5. Visit 4 (Day 21+3) – 2nd vaccination

The second dose of study vaccine should be administrated on Day 21 with an allowed delay of 3 days. All subjects should complete the following study procedures **prior to vaccination**.

(1) Check withdrawal criteria.

(2) Check contraindications to second vaccination.

(3) Conduct a complete physical examination with an exception of body height. Measure and record the oral temperature.

(4) Collect urine samples for pregnancy test (in females of childbearing potential only).

(5) Collect approximately 7 ml of whole blood sample for following central laboratory tests.
- o Immunogenicity tests: IgG and neutralizing antibody titers.

The study vaccine will be administrated intramuscularly (i.m.) in the deltoid of the non-dominant arm. **After vaccination**:

(1) Observe closely during vaccination and at least 2 hours after vaccine administration. An appropriate medical treatment should be readily available in case of a rare anaphylactic reaction following vaccination.

(2) Conduct a telephone follow-up 7 days after vaccine administration.
For subjects in Block C, a 14-day telephone follow-up will also be taken place.

(3) Collect the diary card of 1st vaccination.
The investigator or delegate will verify completion. The information will be transcribed into the CRF. Any unreturned diary cards will be sought from the subjects through telephone call(s) or any other convenient procedure.

(4) Dispense self-evaluation diary card for 2nd vaccination. Instruct the subjects to monitor body temperature and complete the diary correctly.
Any solicited symptoms and local and/or general adverse events occurring during a 7 days post-vaccination period, and any unsolicited symptoms occurring during a 21 days post-vaccination period will be recorded on the diary card. The subjects will be instructed to contact the investigator or their delegates immediately if symptoms should manifest or in case of any signs or symptoms occurring which the subject perceives as serious.
Record concomitant medication(s)/vaccine(s), taken since the previous visit and during vaccination period.

(5) Record and report adverse or serious adverse event if any one has occurred since the previous visit.

5.6. Visit 4a (Day 24± 3) – CPK Monitoring

Creatine kinase (CPK) monitoring will be performed in 10 subjects in Part I for 2nd vaccination on Day 24 with an allowed a time window of 3 day. Whole blood sample for CPK test will be collected and analyzed by local laboratory.

Whether Visit 2a and Visit 4a should be conducted again in Part II based on the Part I results and judged by DSMB.

5.7. Visit 5~7 – Follow-up

There will be three follow-up visits for each participant. The first one will be conducted in the hospital and the other two will be conducted through telephone contact.

5.7.1. Visit 5 (Day 42±3)

Visit 5 will be conducted on Day 42 with a time window of 3 days. The following study procedures will be completed during this period.

(1) Check withdrawal criteria.

(2) Conduct a complete physical examination with an exception of body height. Measure and record the oral temperature.

(3) Collect approximately 8 ml of whole blood sample for following local laboratory tests.
   - Hematological tests: the same as screening visit
   - Biochemistry tests: the same as screening visit

(4) Collect approximately 7 ml of whole blood sample for following central laboratory

tests.

- Immunogenicity tests: IgG and neutralizing antibody titers, and epitope mapping.

(5) Collect the diary card of 2nd vaccination.
The investigator or delegate will verify completion. The information will be transcribed into the CRF. Any unreturned diary cards will be sought from the subjects through telephone call(s) or any other convenient procedure.

(6) Record concomitant medication(s)/vaccine(s), taken since the previous visit.

(7) Record and report adverse or serious adverse event if any one has occurred since the previous visit.

5.7.2. Visit 6 and 7 (Day 126±14 and 210±14) – Telephone follow-up

Visit 6 and Visit 7 will be conducted on Day 126 and 210, respectively. A 14-day time window will be allowed. The following study procedures should be completed and all the information will be collected through telephone call(s).

(1) Check withdrawal criteria (only at Visit 6).

(2) Record concomitant medication(s)/vaccine(s), taken since the previous visit.

(3) Record and report adverse or serious adverse event if any one has occurred since the previous visit.

(4) Complete the Off Study CRF at the final visit.

5.8. Early termination (ET)

For subjects who discontinue this study earlier, a final telephone follow-up may be arranged as soon as possible and all study procedures listed for the final visit (Visit 7) should be completed. However, if a subject is withdrawn at regular return as a reason other than AE, the telephone follow-up could be omitted and no further information has to be collected since last visit.

6. Study Drug

6.1. Treatment assignment

After the informed consent document is signed, all subjects will be assigned the screening number in turn. Only qualified subjects will be uniquely identified by a combination of a 1-digit center number and a 3-digit subject number. The subject numbers will be given in a consecutive order, i.e. initiate from 001 and subsequently follow by 002, 003… etc. Each subject number will randomly correspond to one of the dosing groups below.

- **Group A05:** 0.25 ml per dose (5 μg total protein + adjuvant 150 μg $AlPO_4$)
- **Group B10:** 0.5 ml per dose (10 μg total protein + adjuvant 300 μg $AlPO_4$)

The corresponding study group of individual subject will be recorded in a sealed envelope. It could be opened only before the first vaccination. Both the subject numbers and corresponding dosage should be recorded on the CRF.

Once a number has been assigned, it could not be replaced by other subject.

This study contains two parts, and there are three and one blocks in Part I and Part II, respectively. The blocks will be initiated in turn as well and the randomization will be conducted for each block separately. For the first part, the first subject enrolled will be assigned to Block A, and the subsequent 2 and 7 subjects will be assigned to Block B and C. After that, the rest 50 subjects will be assigned to Block D for the second part of study (Table 6-1).

Randomization scheme will be created by Qualitix Biostatistics & Data Management (BDM). The first eligible subject, 1-001, will be assigned to low-dose group (Group A05) for the consideration of safety. After that, the allocation of study dosage will be conducted through randomization scheme in order to insure that assignment is unbiased and concealed from subjects and investigator staff.

Table 6-1 Number of participants assigned to each block:

Part (Total number)	Block (Total number)	Administration assigned	
		Group	Number of Subjects
Part I (n = 10, in 1 center)	A (n = 1, 1-001)	A05	1
	B (n = 2, 1-002 and 1-003)[*]	A05	1
		B10	1
	C (n = 7, 1-004 to 1-010)[**]	A05	3
		B10	4
Part II (n = 50, in 2 centers)	D (n = 50, 1-011, 1-012… 2-011, 2-012…)[***]	A05	25
		B10	25

[*] Initiate 7 days safety monitoring after 1st vaccination.
[**] Initiate 7 days safety monitoring after 2nd vaccination.
[***] Initiate 14 days safety monitoring after 2nd vaccination.

6.2. Treatment blinding

Not applicable. This is an open-label study.

6.3. Description of study vaccine

This is a Phase I, open-label study. After enrollment, all subjects will be randomized to 2 treatment groups, Group A05 or Group B10, in a 1:1 ratio. Both groups will follow the same visit structure and be administrated 2 doses of the study drug, EV71vac. The information of the study drugs is described as following.

- Development:
  To carry-on the development of a vaccine against EV71 infection, Vaccine Research and Development Center (VRDC) at NHRI has the basic technology through technology transfer agreement with Taiwan CDC, NHRI VRDC has made significant research progress in the serum-free bioprocess for producing cell-cultured-based inactivated whole-virion EV71 candidate vaccine using the roller-bottle technology. The seed virus strain and Vero cells which had been cultivated at Taiwan CDC then

transferred to NHRI VRDC for further development. Vero grown, purified and formaldehyde-inactivated whole virus candidate vaccine was developed and produced in a GCP certified facility of VRDC, NHRI in Taiwan.

- ♦ Active ingredient: Inactivated EV71 whole virion (EV71vac bulk)
- ♦ Dosage form:
  The EV71vac is harvested, ultra-concentrated, purified and inactivated with formalin, and is formularized with aluminum ($AlPO_4$). It contains 20 μg/ml viral protein with adjuvant 600 μg/ml $AlPO_4$.
  The vaccine is formulated as a multi-dose injection, 3 ml sterile suspension per vial, and each vial will be applied for maximum 5 doses at the same time.
- ♦ Dose:
  - o **Group A05:** 0.25 ml per dose (5 μg total protein + adjuvant 150 μg $AlPO_4$)
  - o **Group B10:** 0.5 ml per dose (10 μg total protein + adjuvant 300 μg $AlPO_4$)
- ♦ Pharmacological category: Vaccine
- ♦ Mechanism of action: Preventive vaccination of inactivated EV71 whole virion (EV71vac bulk) to induce antibody against EV71 virus.
- ♦ Appearance: Opalescent liquid
- ♦ Storage: 2-8˚C, do not freeze
- ♦ Manufacture: Vaccine Research and Development Center (VRDC) of National Health Research Institute (NHRI)

Study vaccine will be packaged in appropriate bags labeled with protocol number, name of study center, name of study medication, manufacture date, storage condition and dosing instruction. The warning of “For use in the specified clinical study only” and “Far away from the children” will be labeled.

It is noted that the investigators and/or pharmacists must maintain records of the product’s delivery to the study site, the inventory at the site, and use by each subject, and the return to the sponsor or alternative disposition of unused product(s). These records will include dates, quantities, batch/serial numbers and the unique code numbers assigned to study subjects. Investigators will maintain records that document adequately that subjects were provided the doses specified by the protocol and reconcile all investigational products received from the sponsor. At the time of return to the sponsor, investigators must verify that all unused or partially used drug supplies have been returned by the clinical study subjects and no remaining supplies are in the investigators’ possession.

6.4. Dosage and administration of study vaccine

The immunization schedule consisted of two vaccinations, at least 21-day apart, at the same dose. There are two dosage regiments in this study. Subjects will be injected with 0.25 ml or 0.5 ml vaccine, with aluminium ($AlPO_4$) adjuvant, intramuscularly (i.m.) in the deltoid of the non-dominant arm (Table 6-2).

Table 6-2 Dosage and administration of study vaccine:

Group	Dosage	Content	
		Total protein	Adjuvant $AlPO_4$
A05	0.25 ml	5 µg	150 µg
B10	0.5 ml	10 µg	300 µg

The subjects will be observed closely for at least 2 hours following the administration of study vaccines, with appropriate medical treatment readily available in case of a rare anaphylactic reaction.

6.5. Concomitant treatment

The investigator should instruct the subject to notify the study site about any new medical treatment he/she takes during study period. All vaccines, medications, both permitted and non-permitted, and significant non-drug therapies, such as surgery, physical therapy and blood transfusions, administered during the 210-day study period must be listed on the Concomitant Treatment logs of Case Report Form. Those records may include generic name / trade names (allowed for combination medication only), medical indication, total daily dose, route of administration, start and end dates of treatment

The prior treatment of investigational products, other vaccine(s), immunoglobulins/blood products, immunosuppressants/immunomodulators and acetylsalicylic acid should be check for enrollment criteria (see Section 4.2.2). All treatments administrated 30 days prior to first vaccination should be recorded in the CRF.

All concomitant medications administered for the treatment of an adverse event, at any time, must be recorded as well.

6.5.1. Permitted treatment

Any treatments other than those prohibited in Section 6.5.2 may be administrated during this trial. These may include but not limit:

- ♦ Inhaled and topical steroids
- ♦ Oral contraceptives
- ♦ Vitamins and dietary supplements*

  * The administration of both vitamins and dietary supplements do not have to be recorded in the CRF.

6.5.2. Prohibited treatment

The following medications are prohibited during this clinical study:

(1) The following treatments are prohibited until 30 days after the second vaccination:

- o Other vaccine
- o Immunoglobulins

- o Any blood products
- o Immunosuppressants or other immunomodulators*

  * For corticosteroids, this includes prednisone ≥ 0.5 mg/kg/day or equivalent. Inhaled and topical steroids are allowed.
- o Chronic or long-term (defined as > 14 days) use of acetylsalicylic acid medication

(2) The following treatments are prohibited during the study period:

- o Investigational/non-registered product (including drug, vaccine and invasive medical device)

Subjects who have received these prohibited treatments should be withdrawn from this trial.

6.6. Rescue medication

Rescue medication defined as drugs used to control symptoms that are induced post-vaccination could be administrated based on the investigator's contemplation. Use of rescue medication must be recorded on CRF.

6.7. Drug accountability

Investigators or the designated persons such as clinical research coordinator (CRC) must maintain accurate records of dates and quantities of study product(s) received, batch/serial numbers or other identification number of each delivery, to whom given (subject-by-subject accounting), and accounts of any product accidentally or deliberately destroyed. A Drug Accountability Log must be kept current for individual subject and should contain the following information:

- ♦ The subject's identification to whom the vaccine is administrated.
- ♦ The date(s) of vaccine administrated by the subject.
- ♦ The quantity of vaccine administrated by the subject.

All the used, partially used and unused study vaccines must be retained for the study monitor to confirm the accountability data till study complete. At the conclusion of the study, all those supplies, including empty containers, must be returned to NHRI Vaccine Research and Development Center.

6.8. Treatment exposure and compliance

Not applicable.

7. Assessment Parameters

7.1. Demographics / other baseline characteristics

The demographic and baseline characteristic data for subjects will be collected at screening (Visit 1). The demographics include date of birth, age, gender and ethnicity. Relevant history/conditions include all those present prior to the administration of study vaccine that are listed below:

- relevant medical history,
- all current medical conditions,
- allergy history,
- history of alcohol use,

Whenever possible, diagnoses but not symptoms should be recorded.

7.2. Immunogenicity

The potency of EV71 vaccine ingredient, the inactivated EV71 whole virion (EV71vac bulk), will be evaluated by immunogenicity tests. Blood samples will be collected for these assessments at Visit 2 and Visit 4 prior to each vaccination and at Visit 5. These tests, including EV71 specific IgG antibody titer and EV71 virus neutralization titer, and immune epitope mapping will be conducted at NHRI.

About 7 ml of blood sample should be taken and divided into BD Vacutaineer® SST™ tubes containing silica clot activator polymer and gel for immunogenicity assessments. All blood samples will be collected (without anticoagulant) and stood at room temperature for at least 10 minutes. Clotted blood samples will be centrifuged at 500 x g for 20 minutes at 4˚C, and serum specimens (supernatant) will be transferred into three cryogenic vials (approximately 1000 µl/vial). After that, the blind samples labeled only with specific sample number will be delivered to NHRI under 2~8˚C and stored at -80˚C until processed (refer Sampling guideline in Appendix I).

7.2.1. EV71 specific IgG antibody titer

Determination of EV71 specific IgG antibody titer will be performed with enzyme-linked immunosorbent assay (ELISA). Inactivated EV71 virus was firstly coated on a 96-well microplate and stood for overnight. The microplate will be washed with PBST for one time and blocked with PBS containing 1% of BSA for 2 hours. The microplate will be washed again with PBST for 2 times. Serum specimens will be serially diluted and added into each well. The microplate will be incubated at room temperature for 2 hours and then washed with PBST for 4 times. Detection antibody (goat anti-mouse IgG-Fc antibody HRP, 1:5000) will be added into each well and followed by adding chromogenic substrate (ABST). Absorbance at 405 nm will be then measured.

7.2.2. Neutralizing antibody titers

Determination of EV71 virus neutralization titer is to determine the virus neutralization titer against EV71 virus based on the principle of $TCID_{50}$ assay. Serum specimens will be serially 2-fold diluted and added into each well of the 96-well plate. 100 $TCID_{50}$ of EV71

virus (E59) will be added into each well. The 96-well plate will be the incubated at 37±0.5˚C supplied with 5% CO_2 gas for 4 to 6 days. Virus neutralization titer of a serum will be determined and calculated according to the formula of Reed-Muench method.

7.2.3. Epitope mapping

The human serum samples collected at Visit 2 and Visit 5 (baseline and 42 days after first vaccination) will be used to identify the immunodominant epitopes of EV71 virus.

The overlapping synthetic peptides of the EV71 strain TW/2086/98 VP1, VP2, VP3 and VP4 capsid proteins will be synthesized at Kelowna International Scientific, Inc. There will be a set of 57, 49, 47 and 12 overlapping synthetic peptides spanning the entire sequence of the VP1 to VP4 capsid protein, respectively. Each peptide contains 15 amino acid residues with 10 residues overlapping with the adjacent peptides.

The affinity of antibody to each synthetic peptide will be measured by ELISA. The 96-well plates (Costar) will be coated at 4˚C overnight with 50 µl of individual synthetic peptide or purified virions that will be diluted to 10 µg/ml in coating buffer. Each wells will be blocked by adding 200 µl blocking buffer (5% non-fat milk in PBS). Human serum sample is the primary antibody which will be diluted and added 50 µL per well to coating plate for 2 hours incubation. Shake off the antibody solution and wash 6 times with 1 x PBST for removing primary antibody. Second antibody is anti-human antibody which conjugated with HRP (Horseradish Peroxidase). Second antibody will be added 100 µl per well and incubated for 2 hours. Shake off the antibody solution and wash 6 times with 1 x PBST for removing second antibody. The reaction will be revealed by adding TMB substrate solution (KPL) and incubates at room temperature (RT). The reaction will be stopped by adding H_2SO_4 (1N) and the absorbance will be measured at 450 nm by ELISA reader.

7.3. Safety

7.3.1. Physical examination / body temperature

Complete physical examinations and oral temperature determination should be conducted by investigator at screening, Visit 2 and 4 prior to vaccination, and the last return visit (Visit 5). A complete physical will include the examination of general appearance, HEENT (head, ears, eyes, nose, throat), neck (including thyroid), lymph nodes, skin, cardiovascular, pulmonary, abdomen, neurological system and musculoskeletal/joints.

For two vaccination visits (Visit 2 and Visit 4), body temperature should be measured prior to vaccine administration. Subject with fever defined as oral temperature ≥ 37.5˚C should not receive study vaccine.

Body weight will be measured in indoor clothing, but without shoes at all visits, but body height will be measured only at screening visit. Body mass index (BMI) will be calculated by taking a subject's weight in kilograms (kg) and dividing by their height in meter squared (m^2). A subject with BMI > 35 at screening should be excluded from this study.

It must be recorded when any abnormality has been found out.

7.3.2. Urine pregnancy test

All female subjects except those who have been surgically sterilized (defined as having undergone hysterectomy or bilateral oophorectomy or bilateral salpingectomy; tubal

ligation alone is not considered sufficient) or one year post-menopausal should receive urine pregnancy test. A pregnancy test strip will be used at screening, at Visit 2 prior to randomization and at Visit 4 prior to vaccination. Female with positive result and those who are planning to be pregnant should be withdrawn from this study.

Investigators and their delegates should instruct the females to use medically recognized method of contraception which includes intrauterine contraceptive device; oral contraceptives; diaphragm or condom in combination with contraceptive jelly, cream or foam; Norplant® or DepoProvera® until 2 months after completion of vaccination series.

7.3.3. Selected laboratory safety tests

Fasting blood samples will be collected for hematological and biochemistry examinations at Visit 1, Visit 3 and Visit 5. These tests will be conducted by local laboratory in each center.

7.3.3.1 Hematological examination

The complete blood count (CBC) test includes hemoglobin (Hb), hematocrit (Hct), red blood cells (RBC) count, white blood cells (WBC) count, WBC differential and platelet counts.

Subjects with any significant hematologic abnormality at screening (Visit 1) will not be recruited into this study, and those with any significant abnormality at interim safety screening (Visit 3) should not receive 2nd vaccination.

7.3.3.2 Biochemistry examinations

Biochemistry examinations include alanine transferase (ALT), aspartate transferase (AST), γGT, alkaline phosphatase, total bilirubin, total protein, albumin, BUN, creatinine, uric acid, total cholesterol, triglyceride and fasting plasma glucose (FPG).

Subjects with any significant biochemistry abnormality at screening (Visit 1) will not be recruited into this study, and those with any significant abnormality at interim safety screening (Visit 3) should not receive 2nd vaccination. However, if the level of ALT/AST or creatinine is less than 2.5 x ULN, the test could be conducted again 1 week later and the 2nd dose of EV71 vaccine will be administrated in those with normal results after that.

7.3.4. Self-evaluation (solicited/unsolicited symptoms)

A thermometer and self-evaluation diary card will be dispensed to all subjects after each vaccination. Investigators or their delegates will instruct the subjects to monitor body temperature and complete the all the assessments in the diary every evening.

7.3.4.1 Solicited symptoms

Information of solicited symptoms/AEs will be collected by the subjects in the provided diary cards during a 7-day follow-up period after each vaccination (i.e. day of vaccination and 6 subsequent days), and reported by the investigator team.

A solicited local and general symptoms or AEs are the one whose nature or intensity is consistent with the expected AEs described and listed in Table 7-1.

Table 7-1 Solicited local and general symptoms /adverse events:

Solicited local (at injection site) AEs	Solicited general AEs
o Pain at the injection site	o Fever*
o Tenderness at the injection site	o Nausea/Vomiting
o Redness at the injection site	o Diarrhea
o Swelling at the injection site	o Appetite loss
o Ecchymosis at the injection site	o Headache
	o Myalgia
	o Joint pain
	o Fatigue
	o Shivering

* Oral temperature will be recorded in the evening. Should additional temperature measurements be performed at other times of day, the highest temperature will be recorded.

The oral temperature will be monitored every evening until 6 days after each vaccination. If it has been measured for more than one time, only the highest level should be recorded in the diary card. Subjects with an oral temperature ≥ 37.5˚C will be defined as fever.

7.3.4.2 Unsolicited symptoms

An unsolicited local and general AE or symptoms are the one whose nature or intensity is NOT consistent with the expected AEs described and listed above in this protocol. Information of unsolicited symptoms/AEs will be collected during a 21-day follow-up period after each vaccination (i.e. day of vaccination and 20 subsequent days) and reported at each visit.

7.3.5. Adverse events (AEs)

An adverse event (AE) is defined as any untoward medical occurrence in a subject or clinical investigation subject administered a pharmaceutical product and which does not necessarily have to have a causal relationship with this treatment. An AE can therefore be any unfavorable and unintended sign, symptom, or disease temporally associated with the use of a medicinal product, whether or not considered related to the medicinal product. However, abnormal laboratory values or changes are not automatically reported as adverse events if they are not clinically significant. They will only be recorded as adverse events if a therapeutic action needed or judged by the investigators.

The occurrence of adverse events should be sought by non-directive questioning of the subject at each visit and telephone follow-up (i.e. 7-day/14-day post-vaccination follow-up, and last 2 telephone visits, Visit 6 and Visit 7) during the study. Adverse events also may be detected when they are volunteered by the subject during or between visits or through physical examination, laboratory test or other assessments. All adverse events must be recorded on the Adverse Events CRF with the following information:

1. AE term and description
2. its duration (onset and resolution dates or if continuing at final exam)
3. the severity grade (mild, moderate, severe)
4. whether it constitutes a serious adverse event (SAE)

5. its relationship to the study vaccine (certain/probably-likely/possible/unlikely/ unrelated)
6. action taken treatment required
7. outcome (resolved/not resolve/resolved with residual effect/other)

7.3.5.1 The severity grade of AE and solicited/unsolicited symptoms

The severity of solicited and unsolicited symptoms will be assessed as Table 7-2. It should be scored by subject self if any symptom occurs.

In the diary card, redness / swelling / hematoma at injection site will be recorded as greatest surface diameter in mm, and the maximum oral temperature will be recorded in °C.

Table 7-2 Intensity scales of solicited/unsolicited symptoms:

Adverse Event	Severity grade	Parameter
Solicited local AEs		
Pain at injection site	0	Absent
	1	On touch
	2	When limb is moved
	3	Prevents normal activity
Injection site: Tenderness / Redness / Swelling / Ecchymosis*	0	0 mm
	1	> 0 to ≤ 20 mm
	2	> 20 to ≤ 50 mm
	3	> 50 mm
Solicited general AEs		
Fever**	0	< 37.5°C
	1	≥ 37.5 to ≤ 38.0°C
	2	> 38.0 to ≤ 39.0°C
	3	> 39.0 °C
Nausea/Vomiting	0	Normal
	1	Easily tolerated or 1-2 episodes/day
	2	Interferes with normal activity or > 2 episodes/day
	3	Prevents normal activity, requires outpatient IV hydration
Diarrhea	0	Normal
	1	2-3 loose stools/day
	2	4-5 stools/day
	3	> 6 watery stools/day or requires outpatient IV hydration
Appetite loss / Headache / Myalgia / Joint pain Fatigue / Shivering	0	Normal
	1	Easily tolerated
	2	Interferes with normal activity
	3	Prevents normal activity
Unsolicited AEs		
Unsolicited symptom(s)	0	Normal
	1	Easily tolerated
	2	Interferes with normal activity
	3	Prevents normal activity

* Record greatest surface diameter in mm.
# Record oral temperature in °C. Fever is defined as oral temperature ≥ 37.5°C.

The investigator will make an assessment of the maximum intensity that occurred over the

duration of the event for all other AEs reported during the study period. The assessment will be based on the investigator's clinical judgment. The severity of each AE and SAE recorded in the CRF should be assigned to one of the categories described in Table 7-3.

Table 7-3 Intensity scales of AE:

Severity of AE	Description
1. Mild	Signs or symptoms are being aware of but can be tolerated easily. Signs or symptoms are of minor irritant type and are transient. Symptoms would not require medication or a medical evaluation and there is no loss of time from normal activities.
2. Moderate	Signs or symptoms cause enough discomfort to interfere with usual activities.
3. Severe	Signs or symptoms are incapacitating; subject is unable to do work or usual activities. Signs or symptoms may be of systemic nature or require medical evaluation and/or treatment.

7.3.5.2 The relationship to study vaccine

Table 7-4 The relationship between AE and study vaccine:

Relationship	Description
Certain	A clinical event, including laboratory test abnormality, occurring in a plausible time relationship to drug administration, and which cannot be explained by concurrent disease or other drugs or chemicals. The response to withdrawal of the drug (dechallenge) should be clinically plausible. The event must be definitive pharmacologically or phenomenologically, using a satisfactory rechallenge procedure if necessary.
Probably/likely	A clinical event, including laboratory test abnormality, with a reasonable time sequence to administration of the drug, unlikely to be attributed to concurrent disease or other drugs or chemicals, and which follows a clinically reasonable response on withdrawal (dechallenge). Rechallenge information is not required to fulfill this definition.
Possible	A clinical event, including laboratory test abnormality, with a reasonable time sequence to administration of the drug, but which could also be explained by concurrent disease or other drugs or chemicals. Information on drug withdrawal may be lacking or unclear.
Unlikely	A clinical event, including laboratory test abnormality, with a temporal relationship to drug administration which makes a causal relationship improbable, and in which other drugs, chemicals or underlying disease provide plausible explanations.
Unrelated	A clinical event, including laboratory test abnormality, which is clearly not related to drug administration.

The investigator will make an assessment of the relationship between investigational vaccine and the occurrence of each AE/SAE, including all solicited/unsolicited reactions to

vaccination. The reasonable possibility will be determined based on the investigator's clinical judgment. The causality should be considered as one of the categories described in Table 7-4.

The adverse events should be followed till Day 210 for safety assessments. Each event should be followed until resolution or the event is considered stable. Both regular return and telephone contact will be acceptable.

7.3.6. Serious adverse events (SAEs)

SAE is any experience that suggests a signification hazard, contraindication, side effect or precaution. With respect to human clinical experience this includes any event which:

- results in death,
- is life-threatening,
- requires inpatient hospitalization or prolongation of hospitalization, unless hospitalization is for:
  - routine treatment or monitoring of the studied indication, not associated with any deterioration in condition.
  - elective or pre-planned treatment for a pre-existing condition that is unrelated to the indication under study and has not worsened since the start of study drug
  - treatment on an emergency outpatient basis for an event not fulfilling any of the definitions of a SAE given above and not resulting in hospital admission
  - social reasons and respite care in the absence of any deterioration in the patient's/subject's general condition
- results in persistent of significant disability / incapacity, or
- is a congenital anomaly/birth defect,
- is a significant or important medical event that, based on appropriate medical judgment, may jeopardize the patient/subject or may require intervention to prevent one of the other outcomes listed above.

7.4. Sera cryopreservation

For subjects who agree on ancillary study, the residue samples of immunogenicity test will be preserved in a -80°C freezer for further research.

8. Safety Monitoring

8.1. Serious adverse events reporting requirement and emergency procedures

Whether or not related to the study medication, any adverse event which is fatal or life threatening occurred during the study must be reported promptly (within 24 hours) to Contract Research Organization (CRO), Qualitix, Tel: (02) 3393-1388; Fax: (02)

2351-0607, and Qualitix will inform NHRI on the same day. At the time of the reporting, the following information should be provided if possible: study center, subject number, study phase during which the event occurred, a description of the event, date of onset and current status, outcome, action taken with the investigational vaccine; the reason why the event is classified as serious; and the investigator’s current assessment of the association between the event and investigational vaccine.

Within the required timeframe, the investigator must provide further information on each serious adverse event to Qualitix, and Qualitix will assist investigator to submit to each IRB, ADR reporting center, NHRI and DSMB.

Each recurrent episode, complication, or progression of the initial SAE should be reported as a follow-up to the original episode within 24 hours of the investigator’s receiving the follow-up information. An SAE occurring at a different time interval or otherwise considered completely unrelated to a previous one should be reported separately as a new event.

8.2. Pregnancies

All female subjects will be instructed to use adequate contraceptive precautions until 2 months after completion of the vaccination series (Section 7.3.2). To ensure participant’s safety, each pregnancy in a subject who has received study vaccine must be reported to Qualitix within 24 hours of learning of its occurrence (either from the result of urine pregnancy test or the verbal information). Qualitix should inform NHRI on the same day. The pregnancy outcomes, including spontaneous or voluntary termination, details of the birth, and the presence or absence of any birth defects, congenital abnormalities, or maternal and/or newborn complications, will be followed. Any SAE experienced during pregnancy should be reported as well.

8.3. Data Safety Monitoring Board (DSMB)

The Data and Safety Monitoring Board (DSMB) will act in an advisory capacity to monitor participant safety and evaluate the suitability of second part of study.

8.3.1. DSMB responsibilities

The DSMB responsibilities are to:

- review the study protocol, informed consent documents and plans for data safety and monitoring (refer Section 8.3.2);
- protect the safety of the study participants;
- consider factors external to the study when relevant information becomes available, such as scientific or therapeutic developments that may have an impact on the safety of the participants or the ethics of the trial;
- review interim analyses in accordance with stopping rules, which are clearly defined in Section 8.3.3 and have the approval of the DSMB;
- make recommendations to the NHRI, the Principal Investigator (PI), and, if required, to the Taiwan Food and Drug Administration (TFDA) concerning continuation, termination

or other modifications of the trial based on the observed adverse events/serious adverse events of the vaccination under study;

- make recommendations and assist in the resolution of problems reported by the PI;
- assist the NHRI by commenting on any problems with study conduct and/or data collection.

The DSMB will discharge itself from its duties when the study is complete.

8.3.2. DSMB process

The study could be initiated only after an independent DSMB has reviewed the relevant study materials. 14 days after the last subject of Part 1 has received the second vaccination dose, DSMB will review the initial safety data for each of these 10 study subjects. If no SAE happens prior to interim analysis, the DSMB will recommend continuation of the study recruitment for Part 2.

Throughout the study period in either Part 1 or Part 2, if any vaccine related SAE or serious complaints happen, the PI has to inform DSMB about the event. The DSMB is to make a decision on whether the SAE meets the stopping criteria of the study (see Section 8.4) and conclude if further enrollment of the subjects in the study should be continued or suspended, and will inform the NHRI and PI accordingly. The PI is to inform the NHRI about the study status at the same time. The sponsor is responsible to make the final decision about further enrollment and continuation of the study.

An emergency DSMB meeting can happen any time during the study period.

8.3.3. DSMB meeting format

The meetings could be performed using web/telephone conference. All slides will however be distributed to the individual members of the DSMB prior to the meeting. The DSMB meeting should be scheduled to take place 14 days after the last given injection of subject in Part I. All study data, by the latest 14 days after the last given injection, must be recorded in the CRF and be in-house at Qualitix.

Only DSMB members and the responsible biostatistician will attend this session. The meeting objective is to review data and discuss the data summaries and make recommendations.

8.3.4. Meeting materials

The interim data will be reviewed in a closed session. The Closed Session Report is confidential and marked accordingly. The reports will list and summarize safety data and describe the status of the study. All meeting materials should be sent to the DSMB and the NIA at least 3 days prior to the meeting. The reports are numbered and provided in sealed envelopes within an express mailing package or by secure email as the DSMB prefers.

Copies of reports distributed prior to and during this meeting will be collected by the responsible biostatistician at the end of the Closed Session. Reports distributed to telephone and videoconference participants should be returned or as agreed to the responsible biostatistician for destruction.

8.3.5. Reports from the DSMB

The results from the DSMB will be distributed in a pre specified way without any possibilities for changes. The formal DSMB report should indicate that:

- ♦ the study should continue without any changes, or
- ♦ the study should be stopped* if any of the criteria occur that are defined as study stopping rules in the protocol.

  * This recommendation should be made by formal majority vote. If this should be the case, a specified report will be created and distributed to the NHRI.

Once the report has been finished, the DSMB Chair or NHRI will forward the formal DSMB recommendation to the Principal Investigator. It is the responsibility of the Principal Investigator to distribute the DSMB recommendation to all co-investigators and to ensure that copies are submitted to all the IRBs associated with the study.

8.4. Study stopping criteria

Throughout the study period, if any SAE or serious complain happens, PI is obligated to review subject’s data immediately and make medical judgment if the SAE is vaccine-related or not. If any of the following situations occurs, PI must inform the DSMB and NHRI/CRO and study recruitment should be suspended until further notice.

(1) Any subject with severe local reaction, including ulceration, abscess or necrosis.

(2) Any subject develops laryngospasm, bronchospasm or anaphylaxis.

(3) Two of more subjects develop severe urticaria.

(4) Any vaccine-related SAE during study period.

9. Study Endpoints

9.1. Primary endpoint

- ♦ The percentage, intensity and relationship to vaccination of local and systemic signs and symptoms at a 5-μg and 10-μg dose under the following time frames:
  - o Solicited adverse events: during 7-day follow-up period
  - o Unsolicited adverse events: during 21-day follow-up period
- ♦ The occurrence of overall adverse events and serious adverse event at a 5-μg and 10-μg dose during the entire study period.

9.2. Secondary endpoints

(1) Observed variables from baseline at a 5-μg and 10-μg dose on Day 21 and 42:
  - o Serum IgG titers
  - o Serum neutralizing antibody titers

- o Epitope mapping (only on Day 42)

(2) The change in the laboratory results:

- o Hematology
- o Biochemistry

10. Statistics

10.1. Study cohorts to be evaluated

10.1.1. Total Vaccinated cohort (TVC)

The Total Vaccinated cohort includes all vaccinated subjects who received at least one dose of study medication. For the total analysis of safety, this will include all vaccinated subjects for whom safety data are available. For the total analysis of immunogenicity, this will include vaccinated subjects for whom data concerning immunogenicity endpoint measures are available.

10.1.2. According-To-Protocol (ATP) cohort for analysis of immunogenicity

The ATP cohort for analysis of immunogenicity will include all vaccinated subjects and exclude below subjects:

(1) Subjects are violating major protocol criteria.

(2) Subjects do not have baseline or at least one post-baseline immunogenicity endpoint measures.

10.1.3. According-To-Protocol (ATP) cohort for analysis of safety

The ATP cohort for analysis of safety will include all vaccinated subjects and exclude below subjects:

(1) Subjects are violating major protocol criteria.

(2) Subjects who receive a vaccine not specified or forbidden in the protocol

10.2. Baseline and demographic characteristics

Summary statistics will be provided for demographics (including age, gender, etc.) and for baseline characteristics (including physical examination, vital signs, and laboratory variables). The demographics and baseline characteristics will be presented as counts and percents for the qualitative variables or mean, SD, Min, Max., and range for the quantitative variables as appropriate.

10.3. Analysis of the primary objectives

The primary objective is to evaluate the safety and reactogenicity of the EV71 vaccine at a 5-μg and 10-μg dose.

10.3.1. Variables

The primary endpoints include:

(1) The percentage, intensity and relationship to vaccination of local and systemic signs and symptoms at a 5-μg and 10-μg dose under the following time frames:
   - o Solicited adverse events: during 7-day follow-up period
   - o Unsolicited adverse events: during 21-day follow-up period

(2) The occurrence of overall adverse events and serious adverse event at a 5-μg and 10-μg dose during the entire study period.

10.3.2. Statistical hypothesis, model, and method of analysis

No specific hypothesis is planned to be performed. The main objective is to assess the incidence rate of adverse events. All results will be presented using descriptive statistics.

All solicited and unsolicited adverse events during the study period will be record and analyzed. Adverse events will be coded with MedDRA coding system and will be summarized by system of organ and preferred term. The percentage of subjects with adverse event (solicited and unsolicited) will be tabulated with count, percentage and 95% confidence Interval for each vaccine dose and overall results. The severity and relationship to study medication of adverse events will be summarized as well.

The incidence of serious adverse events will also be summarized; furthermore, the brief summary about serious adverse event will be presented.

10.4. Analysis of secondary objectives

The secondary objectives include:

(1) To evaluate the immunogenicity in terms of IgG titers induced by the EV71 vaccine at a 5-μg and 10-μg dose.

(2) To evaluate the immunogenicity in terms of neutralizing antibody titers from baseline induced by the EV71 vaccine at a 5-μg and 10-μg dose.

(3) To identify the immunodominant epitopes of EV71 virus by human serum.

10.4.1. Variables

The secondary endpoints include:

(1) Observed variables from baseline at a 5-μg and 10-μg dose on Day 21 and 42:
   - o Serum IgG titers
   - o Serum neutralizing antibody titers
   - o Epitope mapping (only on Day 42)

The immunogenicity parameters include serum IgG titers and neutralizing antibody titers on baseline, Day 21 and Day 42, and immune epitope mapping of EV71vac will be conducted on baseline and Day 42.

The serum IgG and neutralizing antibody titers on baseline, Day 21 and Day 42 will be summarized descriptively and the change from baseline will be tested by paired T-test or

Wilcoxon signed rank test under significance level 0.05. The results of immune epitope mapping will be summarized descriptively as well.

(2) The change in the laboratory results:

- Hematology
- Biochemistry

The analysis of laboratory includes hematology and biochemistry.

Hematology tests include CBC, such as Hb, Hct, RBC count, WBC count, WBC differential and platelet count; and biochemistry tests include ALT, AST, γGT, alkaline phosphatase, total bilirubin, total protein, albumin, CPK, BUN, creatinine, uric acid, total cholesterol, triglyceride and glucose (fasting).

The descriptive summary results of laboratory for the baseline, each study visit, end of visit, the change from baseline to end of visit will be presented and paired T-test or Wilcoxon signed rank test will be used to test change from baseline under significance level 0.05.

10.4.2. Safety

The evaluated variables for safety include vital signs and physical examination.

(1) Vital signs

The summary of vital signs includes systolic/diastolic blood pressure, heart rate and body temperature. The descriptive summary results for the baseline, each study visit, and end of visit, the change from baseline to end of visit will be presented, and paired T-test or Wilcoxon signed rank test will be used to test change from baseline under significance level 0.05.

(2) Physical examination

The physical examination will be summarized descriptively by visit. Besides, the transition state of physical examination from baseline to end of visit will be summarized as well.

10.5. Handling of missing data

For the missing data of each endpoint, no method will be performed for missing data handling.

10.6. Sample size and power considerations

Not applicable.

10.7. Planned interim analysis

An interim safety analysis will be conducted 14 days after the second vaccination of Block C. The second part of study could be initiated only after the interim report has been reviewed and approved by DSMB.

11. Compliance of Ethics

11.1. Ethical conduct of the study

The study will be performed in accordance with the protocol, ICH Harmonized Tripartite Guidelines for Good Clinical Practice (GCP), applicable local regulations and recommendations guiding physicians in biomedical research involving human subjects adopted by the 18th World Medical Assembly, Helsinki, Finland, 1964 and later revisions (Appendix II).

11.2. Institutional Review Board (IRB)/ethics committee (EC)

The Principal Investigator agrees to provide the IRB or EC with all appropriate material, including the informed consent document. The trial will not be initiated until appropriate IRB approval of the protocol and the informed consent document, and the sponsor (NHRI, Vaccine Research and Development Center) has received copies of the submitted documents and the approval letter. Appropriate reports on the progress of the study by the Principal Investigator will be made to the IRB and the sponsor in a timely manner in accordance with applicable government regulations and in agreement with policy established by the sponsor. In addition, investigators are responsible for reporting serious adverse events, as defined in the protocol, to IRB/EC at each center according to applicable regulation.

11.3. Informed consent process and subject information

A properly executed written informed consent in Chinese language shall be obtained from each subject prior to entering the subject into the trial or prior to performing any procedure that involves risk to the subject. Each investigator shall provide a copy of the IRB-approved informed consent to every subject [or the subject's legal representative, if appropriate] and a signed copy shall be maintained in the subject's record file. Attention is directed to the basic elements that are required to be incorporated into the informed consent under International Conference on Harmonization (ICH) Guideline for Good Clinical Practice (GCP) and in accordance with the latest version of the Declaration of Helsinki (Adopted by the 18th WMA General Assembly Helsinki, Finland, June 1964 and amended by the 59th WMA General Assembly, Seoul, South Korea, October 2008). The information includes experimental setting, trial objectives, possible benefits, side effects and dangers of participation in the trial, currently available alternative procedures or treatment regimens, the rights and responsibilities of the trial subject, and other information that is relevant to the subject's decision to participate.

12. Insurance and Indemnity

All subjects will be insured by NHRI, Vaccine Research and Development Center against complications of any kind, which are directly caused by the administration of the investigational vaccine or the study procedures. Information regarding insurance and indemnity will be provided to the investigators by NHRI, Vaccine Research and Development Center prior to the initiation of this trial.

13. Sources Documents

To enable evaluations and/or audits from health authorities or NHRI, Vaccine Research and Development Center (VRDC), investigators agree to keep records, including the identity of all participating subjects, all original signed Informed Consent Forms (ICFs), copies of all CRFs and detailed records of medication disposition. The medical institution should retain the records of the trial subjects forever. Investigators and sponsor retain the subject identification and all trial-related documents codes for the regulatory authorities and the SOP of the sponsor requirement.

All information provided to the investigators by sponsor including pre-clinical data, protocols, CRFs should be kept confidential and confined to the clinical personnel involved in conducting the study or authorized representatives of appropriate health/regulatory authorities. Identity and hospital records of each subject are also kept confidential except for authorized personnel.

14. Case Report Form

NHRI's policy of that study data must be verifiable to the source data, which necessitates access to all original recordings, laboratory reports, and subjects' records. Investigators must therefore agree to allow access to subjects' records and source data by NHRI, CRO (Qualitix Clinical Research) and Health Authorities personnel. The subjects (or their legal representatives) must also allow access to the subjects' medical records, and they will be informed of this and will be signing their agreement when giving informed consent. Correction to data on CRFs may be made only by putting a line through the incorrect data and writing the correct values, allowing the original text to remain legible. Each correction must be initialed and dated by the person making the change. If corrections are made after review and signature by the investigator, he must be made aware of the changes.

15. Control and Assurance of Study Quality

15.1. Assignment of Site Monitor

All aspects of the study will be conducted under ICH and GCP guidelines. Qualified individuals designated by the sponsor will monitor it. Monitoring will be conducted according to GCP and standard operating procedures for compliance with applicable government regulations. Investigators agree to allow these monitors access to the clinical supplies, dispensing, and test drug storage areas and to the clinical files of the study subjects, and if requested, agrees to assist the monitors.

15.2. Data Quality Assurance

The study is monitored by regular site visits and telephone calls to the investigators by NHRI, Vaccine Research and Development Center or CRO (Qualitix) representatives. During site visits, the monitor will review original subject records, drug accountability records, and study file. The monitor will also observe study procedures and discuss any

problems with the investigator.

16. Protocol Deviations

All deviations from the protocol should be reported to NHRI Vaccine Research and Development Center. This includes but is not limited to the following:

1. Subjects who entered the study even though they did not satisfy the entry criteria;
2. Subjects who developed withdrawal criteria during the study but were not withdrawn;
3. Subjects who received the wrong treatment or incorrect dose;
4. Subjects who received concomitant treatment in an incorrect manner.

Additionally, exceptions or deviations that could conceivably affect the collection or interpretation of data related to safety or efficacy assessments should also be reported. Examples include subject noncompliance with medication, surreptitious use of medications prohibited by the protocol, inability or unwillingness to accurately report adverse events or concomitant medication use.

17. Protocol Amendments

Only the sponsor may modify the protocol. Amendments to the protocol will only be made after consultation and agreement between sponsor and investigator. All amendments that have an impact on subject risk or the study objectives, or require revision of the informed consent document, must receive approval from the IRB prior to their implementation.

18. Termination of Study

National Health Research Institutes, Vaccine Research and Development Center reserves the right to discontinue this trial at any time. The reasons for such action include, but are not limited to:

- ♦ Safety concerns
- ♦ The investigator does not comply with the protocol, Good Clinical Practice (GCP) and/or any contract between the investigator and National Health Research Institutes, Vaccine Research and Development Center, including CROs and subsidiaries hereof.
- ♦ For failure to meet expected enrollment goals
- ♦ The required number of subjects for the study has already been recruited.

19. Retention of Records and Confidentiality

Clinical investigators are responsible to keep study information (e.g. the original signed Informed Consent, copies of CRFs, and detailed records of medication disposition) pertaining to the subject’s identity in a confidential manner. When the need arises, only the health authorities or NHRI can have access to this information.

20. Use of Information and Publication

Neither any part of the study protocol or its amendments nor information given by NHRI to the clinical investigators for the purpose of evaluating and/or performing the study shall be disclosed to any third party without prior written consent from NHRI. Investigators are obliged to provide NHRI with copies of all data derived from the study. Except for required by law, only NHRI has the sole right to disclose the study information to other physicians and regulatory agencies.

21. References

1. Cardosa MJ, Perera D, Brown BA, *et al*. Molecular epidemiology of human enterovirus 71 strains and recent outbreaks in the Asia-Pacific region: comparative analysis of the VP1 and VP4 genes. *Emerg Infect Dis*. 2003 Apr;9(4):461-8.
2. Shimizu H, Utama A, Yoshii K, *et al*. Enterovirus 71 from fatal and nonfatal cases of hand, foot and mouth disease epidemics in Malaysia, Japan and Taiwan in 1997-1998. *Jpn J Infect Dis*. 1999 Feb;52(1):12-5.
3. Wang CY, Li Lu F, Wu MH, *et al*. Fatal coxsackievirus A16 infection. *Pediatr Infect Dis J*. 2004 Mar;23(3):275-6.
4. Deaths among children during an outbreak of hand, foot, and mouth disease — Taiwan, Republic of China, April–July 1998. *MMWR Morb Mortal Wkly Rep*. 1998;47:629-32.
5. Lin TY, Twu SJ, Ho MS, *et al*. Enterovirus 71 outbreaks, Taiwan: occurrence and recognition. *Emerg Infect Dis*. 2003 Mar;9(3):291-3.
6. Kuo HSea. Communicable Diseases of Interest to the Public: Enterovirus. Center for Disease Control, Taiwan. 2005 Annual Report. 2005:53-5.
7. Huang CC, Liu CC, Chang YC, *et al*. Neurologic complications in children with enterovirus 71 infection. *N Engl J Med*. 1999 Sep 23;341(13):936-42.

Appendix I Immunogenicity sampling guideline (central laboratory)

Sampling time:

Blood sample will be collected for immunogenicity tests at following time points.

(1) Visit 2 (Day 0) prior to each vaccination

(2) Visit 4 (Day 21) prior to each vaccination

(3) Visit 5 (Day 42) at the same time as the other hematology/biochemistry tests

Vaccination time and sampling times should be recorded accurately on the Sampling Log.

Blood collection and handling:

(1) Collect approximately 7 ml whole blood sample (without anticoagulant).

(2) Divide into BD Vacutainer® SST™ tubes containing silica clot activator polymer and gel.

(3) Let blood stand at room temperature for at least 10 minutes.

(4) Centrifuge the clotted blood samples at 500 x g for 20 minutes at 4˚C.

(5) Transfer the serum specimens (supernatant) into 3 cryogenic vials (approximately 1000 µl/vial).

(6) Store the cryogenic vials at -80˚C freezer until shipment (i.e. last subject at each site completes Visit 5).

Sample shipment

All blood samples at each site will be delivered to central laboratory, NHRI VRDC, under 2~8˚C after the last subject completes Visit 5. After shipment, the samples will be stored at -80˚C freezer at NHRI until further testing.

Blinded analysis for central laboratory:

All immunogenicity tests will be analyzed through the following blinded procedures in the central laboratory.

(1) Blind samples: The samples will only be labeled with specific sample number and all blood samples will be shipped at the same time. No subject information will be explored on the sample labels.

(2) Blinded analyzer: The immunogenicity assessments will be performed by NHRI. However, all treatment information will be blinded among the central laboratory.

Appendix II

Initiated: 1964
Original: English

WORLD MEDICAL ASSOCIATION DECLARATION OF HELSINKI
Ethical Principles for Medical Research Involving Human Subjects

Adopted by the
18th WMA General Assembly, Helsinki, Finland, June 1964
and amended by the
29th WMA General Assembly, Tokyo, Japan, October 1975
35th WMA General Assembly, Venice, Italy, October 1983
41st WMA General Assembly, Hong Kong, September 1989
48th WMA General Assembly, Somerset West, Rep. of South Africa, Oct. 1996
52nd WMA General Assembly, Edinburgh, Scotland, October 2000
53rd WMA General Assembly, Washington USA, 2002 (Note of Clarification)
55th WMA General Assembly, Tokyo, Japan 2004 (Note of Clarification)
59th WMA General Assembly, Seoul, South Korea, October 2008

(1) INTRODUCTION

1. The World Medical Association (WMA) has developed the Declaration of Helsinki as a statement of ethical principles for medical research involving human subjects, including research on identifiable human material and data.
   The Declaration is intended to be read as a whole and each of its constituent paragraphs should not be applied without consideration of all other relevant paragraphs.
2. Although the Declaration is addressed primarily to physicians, the WMA encourages other participants in medical research involving human subjects to adopt these principles.
3. It is the duty of the physician to promote and safeguard the health of patients, including those who are involved in medical research. The physician's knowledge and conscience are dedicated to the fulfilment of this duty.
4. The Declaration of Geneva of the World Medical Association binds the physician with the words, "The health of my patient will be my first consideration," and the International Code of Medical Ethics declares that, "A physician shall act only in the patient's interest when providing medical care which might have the effect of weakening the physical and mental condition of the patient."
5. Medical progress is based on research that ultimately must include studies involving human subjects. Populations that are underrepresented in medical research should be provided appropriate access to participation in research.

6. In medical research involving human subjects, the well-being of the individual research subject must take precedence over all other interests.
7. The primary purpose of medical research involving human subjects is to understand the causes, development and effects of diseases and improve preventive, diagnostic and therapeutic interventions (methods, procedures and treatments). Even the best current interventions must be evaluated continually through research for their safety, effectiveness, efficiency, accessibility and quality.
8. In medical practice and in medical research, most interventions involve risks and burdens.
9. Medical research is subject to ethical standards that promote respect for all human subjects and protect their health and rights. Some research populations are particularly vulnerable and need special protection. These include those who cannot give or refuse consent for themselves and those who may be vulnerable to coercion or undue influence.
10. Physicians should consider the ethical, legal and regulatory norms and standards for research involving human subjects in their own countries as well as applicable international norms and standards. No national or international ethical, legal or regulatory requirement should reduce or eliminate any of the protections for research subjects set forth in this Declaration.

(2) BASIC PRINCIPLES FOR ALL MEDICAL RESEARCH

11. It is the duty of physicians who participate in medical research to protect the life, health, dignity, integrity, right to self-determination, privacy, and confidentiality of personal information of research subjects.
12. Medical research involving human subjects must conform to generally accepted scientific principles, be based on a thorough knowledge of the scientific literature, other relevant sources of information, and adequate laboratory and, as appropriate, animal experimentation. The welfare of animals used for research must be respected.
13. Appropriate caution must be exercised in the conduct of medical research that may harm the environment.
14. The design and performance of each research study involving human subjects must be clearly described in a research protocol. The protocol should contain a statement of the ethical considerations involved and should indicate how the principles in this Declaration have been addressed. The protocol should include information regarding funding, sponsors, institutional affiliations, other potential conflicts of interest, incentives for subjects and provisions for treating and/or compensating subjects who are harmed as a consequence of participation in the research study. The protocol should describe arrangements for post-study access by study subjects to interventions identified as beneficial in the study or access to other appropriate care or benefits.
15. The research protocol must be submitted for consideration, comment, guidance and approval to a research ethics committee before the study begins. This committee must be independent

of the researcher, the sponsor and any other undue influence. It must take into consideration the laws and regulations of the country or countries in which the research is to be performed as well as applicable international norms and standards but these must not be allowed to reduce or eliminate any of the protections for research subjects set forth in this Declaration. The committee must have the right to monitor ongoing studies. The researcher must provide monitoring information to the committee, especially information about any serious adverse events. No change to the protocol may be made without consideration and approval by the committee.

16. Medical research involving human subjects must be conducted only by individuals with the appropriate scientific training and qualifications. Research on patients or healthy volunteers requires the supervision of a competent and appropriately qualified physician or other health care professional. The responsibility for the protection of research subjects must always rest with the physician or other health care professional and never the research subjects, even though they have given consent.
17. Medical research involving a disadvantaged or vulnerable population or community is only justified if the research is responsive to the health needs and priorities of this population or community and if there is a reasonable likelihood that this population or community stands to benefit from the results of the research.
18. Every medical research study involving human subjects must be preceded by careful assessment of predictable risks and burdens to the individuals and communities involved in the research in comparison with foreseeable benefits to them and to other individuals or communities affected by the condition under investigation.
19. Every clinical trial must be registered in a publicly accessible database before recruitment of the first subject.
20. Physicians may not participate in a research study involving human subjects unless they are confident that the risks involved have been adequately assessed and can be satisfactorily managed. Physicians must immediately stop a study when the risks are found to outweigh the potential benefits or when there is conclusive proof of positive and beneficial results.
21. Medical research involving human subjects may only be conducted if the importance of the objective outweighs the inherent risks and burdens to the research subjects.
22. Participation by competent individuals as subjects in medical research must be voluntary. Although it may be appropriate to consult family members or community leaders, no competent individual may be enrolled in a research study unless he or she freely agrees.
23. Every precaution must be taken to protect the privacy of research subjects and the confidentiality of their personal information and to minimize the impact of the study on their physical, mental and social integrity.
24. In medical research involving competent human subjects, each potential subject must be adequately informed of the aims, methods, sources of funding, any possible conflicts of

interest, institutional affiliations of the researcher, the anticipated benefits and potential risks of the study and the discomfort it may entail, and any other relevant aspects of the study. The potential subject must be informed of the right to refuse to participate in the study or to withdraw consent to participate at any time without reprisal. Special attention should be given to the specific information needs of individual potential subjects as well as to the methods used to deliver the information. After ensuring that the potential subject has understood the information, the physician or another appropriately qualified individual must then seek the potential subject's freely-given informed consent, preferably in writing. If the consent cannot be expressed in writing, the non-written consent must be formally documented and witnessed.

25. For medical research using identifiable human material or data, physicians must normally seek consent for the collection, analysis, storage and/or reuse. There may be situations where consent would be impossible or impractical to obtain for such research or would pose a threat to the validity of the research. In such situations the research may be done only after consideration and approval of a research ethics committee.
26. When seeking informed consent for participation in a research study the physician should be particularly cautious if the potential subject is in a dependent relationship with the physician or may consent under duress. In such situations the informed consent should be sought by an appropriately qualified individual who is completely independent of this relationship.
27. For a potential research subject who is incompetent, the physician must seek informed consent from the legally authorized representative. These individuals must not be included in a research study that has no likelihood of benefit for them unless it is intended to promote the health of the population represented by the potential subject, the research cannot instead be performed with competent persons, and the research entails only minimal risk and minimal burden.
28. When a potential research subject who is deemed incompetent is able to give assent to decisions about participation in research, the physician must seek that assent in addition to the consent of the legally authorized representative. The potential subject's dissent should be respected.
29. Research involving subjects who are physically or mentally incapable of giving consent, for example, unconscious patients, may be done only if the physical or mental condition that prevents giving informed consent is a necessary characteristic of the research population. In such circumstances the physician should seek informed consent from the legally authorized representative. If no such representative is available and if the research cannot be delayed, the study may proceed without informed consent provided that the specific reasons for involving subjects with a condition that renders them unable to give informed consent have been stated in the research protocol and the study has been approved by a research ethics committee. Consent to remain in the research should be obtained as soon as possible from the subject or a legally authorized representative.

30. Authors, editors and publishers all have ethical obligations with regard to the publication of the results of research. Authors have a duty to make publicly available the results of their research on human subjects and are accountable for the completeness and accuracy of their reports. They should adhere to accepted guidelines for ethical reporting. Negative and inconclusive as well as positive results should be published or otherwise made publicly available. Sources of funding, institutional affiliations and conflicts of interest should be declared in the publication. Reports of research not in accordance with the principles of this Declaration should not be accepted for publication.

(3) ADDITIONAL PRINCIPLES FOR MEDICAL RESEARCH COMBINED WITH MEDICAL CARE

31. The physician may combine medical research with medical care only to the extent that the research is justified by its potential preventive, diagnostic or therapeutic value and if the physician has good reason to believe that participation in the research study will not adversely affect the health of the patients who serve as research subjects.
32. The benefits, risks, burdens and effectiveness of a new intervention must be tested against those of the best current proven intervention, except in the following circumstances:
    - The use of placebo, or no treatment, is acceptable in studies where no current proven intervention exists; or
    - Where for compelling and scientifically sound methodological reasons the use of placebo is necessary to determine the efficacy or safety of an intervention and the patients who receive placebo or no treatment will not be subject to any risk of serious or irreversible harm. Extreme care must be taken to avoid abuse of this option.
33. At the conclusion of the study, patients entered into the study are entitled to be informed about the outcome of the study and to share any benefits that result from it, for example, access to interventions identified as beneficial in the study or to other appropriate care or benefits.
34. The physician must fully inform the patient which aspects of the care are related to the research. The refusal of a patient to participate in a study or the patient's decision to withdraw from the study must never interfere with the patient-physician relationship.
35. In the treatment of a patient, where proven interventions do not exist or have been ineffective, the physician, after seeking expert advice, with informed consent from the patient or a legally authorized representative, may use an unproven intervention if in the physician's judgement it offers hope of saving life, re-establishing health or alleviating suffering. Where possible, this intervention should be made the object of research, designed to evaluate its safety and efficacy. In all cases, new information should be recorded and, where appropriate, made publicly available.
