# Supplementary material for: Formalin-Inactivated EV71 Vaccine Candidate Induced Cross-Neutralizing Antibody against Subgenotypes B1, B4, B5 and C4A in Adult Volunteers
Source: PLoS One. 2013 Nov 21;8(11):e79783. doi: 10.1371/journal.pone.0079783 (PMC3836818; doi:10.1371/journal.pone.0079783)
Supplement: File S1 — This file describes the Supplementary Materials of Formalin-inactivated EV71 vaccine candidate induced cross-neutralizing antibody against subgenotypes B1, B4, B5 and C4A in adult volunteers. The contents include: Table S1. Summary of pre- and post-vaccination immune responses from individual subjects. Table S2. Summary of EV71 B genotypes-specific virus neutralizing antibody titers obtained from subjects immunized with the EV71 vaccine. Table S3. Summary of EV71 C4 subgenotypes-specific virus neutralizing antibody titer obtained from human sera immunized with EV71 vaccine. Figure S1. Virus neutralizing antibody responses of young adults immunized with EV71vac against subgenotype B4 (vaccine strain). Figure S1. Virus neutralizing antibody responses of young adults immunized with EV71vac against subgenotype B4 (vaccine strain). Panels A and B show the neutralization titers of sera obtained from volunteers immunized twice with either a 5- or 10-µg dose, respectively. Thirty healthy adults were enrolled in each group. Each spot represents the neutralizing antibody titer for individual sera. The bar corresponds to the geometric mean titer (GMT) of each set of sera. (DOC) [file pone.0079783.s002.doc]

**Supplementary Materials**

**Formalin-inactivated EV71 vaccine candidate induced cross-neutralizing antibody against subgenotypes B1, B4, B5 and C4A in adult volunteers**

Ai-Hsiang Chou^a,1^, Chia-Chyi Liu^a,1^, Jui-Yuan Chang^a^, Renee Jiang^a^, Yi-Chin Hsieh^a^, Amanda Tsao^a^, Chien-Long Wu^a^, Ju-Lan Huang^a^, Chang-Phone Fung^b^, Szu-Min Hsieh^c^, Ya-Fang Wang^a^, Jen-Ren Wang^a^, Mei-Hua Hu^a^, Jen-Ron Chiang^d^, Ih-Jen Su^a^ and Pele Chong^a,e,*^

^a^Vaccine R&D Center, National Institute of Infectious Diseases and Vaccinology, National Health Research Institutes, Zhunan Town, Miaoli County, Taiwan 350. ^b^Veterans General Hospital Taipei, Taipei, Taiwan. ^c^National Taiwan University Hospital, Taipei, Taiwan. ^d^Taiwan CDC, Taipei, Taiwan. ^e^Graduate Institute of Immunology, China Medical University, Taichung, Taiwan.

^1^ These authors contributed equally to this work.

* Corresponding author: Pele Chong, Vaccine R&D Center, National Institute of Infectious Diseases and Vaccinology, National Health Research Institutes, Zhunan Town, Miaoli County, Taiwan 350. 886-37-246 166, ext 37800; Email: [pelechong@nhri.org.tw](mailto:pelechong@nhri.org.tw) (PC).

Table S1. Summary of pre- and post-vaccination immune responses from individual subjects

| Subject # | Day 0 | | | | Day 42 | | | |
| --- | --- | --- | --- | --- | --- | --- | --- | --- |
|  | IgG reactivity with | Neutralization titers against human enterovirus | | | IgG reactivity with | Neutralization titers against human enterovirus | | |
|  | EV71 | B4 | C2 | CVA16 | EV71 | B4 | C2 | CVA16 |
| 1 | 1000 | **100** | 10 | **<8** | 64000 | **2004.75** | 177.83 | **10** |
| 2 | 1000 | **15.85** | <8 | **12.59** | 128000 | **709.63** | 100 | **13.51** |
| 3 | 4000 | **44.67** | 12.59 | **12.59** | 128000 | **1592.43** | 177.83 | **15.85** |
| 4 | 2000 | **10** | <8 | **39.81** | 32000 | **5677.01** | 177.83 | **501.19** |
| 5 | 1000 | **<8** | <8 | **<8** | 32000 | **199.53** | <8 | **31.62** |
| 6 | 2000 | **<8** | <8 | **<8** | 16000 | **25.12** | 11.22 | **39.81** |
| 7** | <1000 | **10** | <8 | **13.51** | **NS** | **NS** |  | **NS** |
| 8 | 1000 | **31.62** | 11.22 | **<8** | 8000 | **294.6** | 22.39 | **<8** |
| 9 | 4000 | **89.13** | <8 | **11.22** | 16000 | **632.46** | 44.67 | **11.22** |
| 10 | 1000 | **11.22** | <8 | **<8** | 4000 | **316.23** | 15.85 | **11.22** |
| 11 | 1000 | **<8** | <8 | **<8** | 8000 | **316.23** | <8 | **<8** |
| 12* | <1000 | **<8** | <8 | **<8** | 8000 | **1004.75** | <8 | **<8** |
| 13 | 2000 | **427.34** | <8 | **<8** | 8000 | **1419.25** | <8 | **19.95** |
| 14* | <1000 | **<8** | <8 | **<8** | 16000 | **50.12** | <8 | **25.12** |
| 15*** | 4000 | **125.89** | <8 | **<8** | 8000 | **355.66** | <8 | **<8** |
| 16 | 1000 | **12.59** | <8 | **<8** | 32000 | **1596.21** | 19.95 | **<8** |
| 17 | 1000 | **<8** | <8 | **10** | 16000 | **177.83** | <8 | **50.12** |
| 18**** | <1000 | **<8** | <8 | **<8** | 8000 | **15.85** | <8 | **<8** |
| 19 | 1000 | **15.85** | <8 | **<8** | 8000 | **2009.51** | 11.22 | **<8** |
| 20 | 2000 | **39.81** | <8 | **<8** | 8000 | **709.63** | <8 | **<8** |
| 21 | 1000 | **31.62** | <8 | **<8** | 32000 | **2009.51** | <8 | **<8** |
| 22# | <1000 | **<8** | <8 | **<8** | 8000 | **44.67** | <8 | **<8** |
| 23 | 1000 | **12.59** | <8 | **<8** | 8000 | **502.38** | <8 | **<8** |
| 24 | 2000 | **44.67** | <8 | **<8** | 8000 | **502.38** | <8 | **<8** |
| 25 | <1000 | **12.59** | <8 | **<8** | 32000 | **428.35** | <8 | **11.22** |
| 26 | 2000 | **125.89** | <8 | **<8** | 8000 | **1264.91** | <8 | **<8** |
| 27 | 2000 | **12.59** | <8 | **44.67** | 8000 | **632.46** | <8 | **31.62** |
| 28 | 1000 | **39.81** | <8 | **12.59** | 32000 | **1596.21** | <8 | **10** |
| 29 | 1000 | **63.1** | <8 | **<8** | 32000 | **1419.25** | <8 | **<8** |
| 30 | 1000 | **39.81** | <8 | **<8** | 16000 | **5059.64** | 13.51 | **<8** |
| 31**** | <1000 | **<8** | <8 | **<8** | 8000 | **<8** | <8 | **<8** |
| 32 | 1000 | **28.18** | <8 | **<8** | 8000 | **632.46** | <8 | **<8** |
| 33 | 2000 | **22.39** | <8 | **<8** | 32000 | **1419.25** | <8 | **<8** |
| 34* | <1000 | **<8** | <8 | **<8** | 32000 | **2009.51** | <8 | **25.12** |
| 35 | 1000 | **<8** | <8 | **<8** | 32000 | **1264.91** | 11.22 | **11.22** |
| 36 | 1000 | **50.12** | <8 | **<8** | 4000 | **199.53** | <8 | **<8** |
| 37 | 1000 | **<8** | <8 | **<8** | 8000 | **63.1** | <8 | **19.85** |
| 38 | 2000 | **<8** | <8 | **<8** | 8000 | **1264.91** | <8 | **31.62** |
| 39* | <1000 | **<8** | <8 | **<8** | 4000 | **709.63** | <8 | **19.95** |
| 40 | 2000 | **<8** | <8 | **<8** | 8000 | **1004.75** | <8 | **15.85** |
| 41 | 2000 | **22.39** | <8 | **<8** | 32000 | **1264.91** | <8 | **10** |
| 42 | 1000 | **<8** | <8 | **<8** | 8000 | **125.89** | <8 | **11.22** |
| 43* | <1000 | **<8** | <8 | **<8** | 4000 | **502.38** | <8 | **<8** |
| 44* | <1000 | **<8** | <8 | **<8** | 8000 | **711.31** | <8 | **63.1** |
| 45 | 1000 | **<8** | <8 | **<8** | 8000 | **79.43** | <8 | **25.12** |
| 46 | 1000 | **<8** | <8 | **<8** | 4000 | **199.53** | <8 | **22.39** |
| 47 | <1000 | **11.22** | <8 | **<8** | 4000 | **1264.91** | <8 | **<8** |
| 48 | 1000 | **63.1** | <8 | **<8** | 16000 | **798.1** | <8 | **<8** |
| 49 | 4000 | **<8** | <8 | **<8** | 16000 | **177.83** | <8 | **15.85** |
| 50 | 1000 | **<8** | <8 | **<8** | 4000 | **12.59** | <8 | **<8** |
| 51 | 2000 | **22.39** | <8 | **<8** | 32000 | **2529.82** | <8 | **<8** |
| 52 | 1000 | **44.67** | <8 | **<8** | 8000 | **1419.25** | <8 | **<8** |
| 53 | 1000 | **<8** | <8 | **<8** | 8000 | **89.13** | <8 | **<8** |
| 54* | <1000 | **<8** | <8 | **<8** | 8000 | **399.05** | <8 | **<8** |
| 55 | 4000 | **19.95** | <8 | **44.67** | 16000 | **798.1** | <8 | **39.81** |
| 56*** | 8000 | **19.95** | <8 | **15.85** | 8000 | **63.1** | <8 | **11.22** |
| 57 | 1000 | **<8** | <8 | **<8** | 16000 | **89.13** | <8 | **53.8** |
| 58 | 1000 | **<8** | <8 | **15.85** | 8000 | **63.1** | <8 | **25.12** |
| 59 | <1000 | **12.59** | <8 | **<8** | 8000 | **177.83** | <8 | **<8** |
| 60 | 1000 | **<8** | <8 | **<8** | 32000 | **3192.42** | <8 | **214.18** |

The subjects immunized with 5 µg of EV71vac are highlighted.

* Subjects most likely not have been exposed to and/or infected with EV71.

** Subject #7 withdrew after the first visit. Blood samples from Day 20 and 42 are not available.

*** Subjects #15 and #56 failed to increase their IgG titers were immunized with 5- and 10-µg of EV71vac, respectively.

**** Subject #31 had an Nt of 20 after the first 10-µg dose of EV71vac but Nt dropped to <8 after 2 doses.

***** Subject #18 had an Nt <8 after one 5-µg injection.

Table S2. Summary of EV71 B genotypes-specific virus neutralizing antibody titers obtained from subjects immunized with the EV71 vaccine.

| EV71 virus | Group |  | | Post 1^st^-vaccination | | Post 2^nd^-vaccination | |
| --- | --- | --- | --- | --- | --- | --- | --- |
|  |  | Pre-vaccination | | >4-fold increase Nt | Neutralization titer (GMT) | >4-fold increase Nt | Neutralization titer (GMT) |
| EV71/0204/TW86  (B1) | A | <1:8 | 11/30 (36.7%) | 9/11 (81.8%) | 92.2 | 10/11 (90.9%) | 86.5 |
|  |  | >1:8 | 19/30 (63.3%) | 17/19 (89.5%) | 870.7 | 16/19 (84.2%) | 676.1 |
|  | B | <1:8 | 11/30 (36.7%) | 8/10 ^a^, (80.0%) | 130.6 | 8/11 (72.7%) | 56.7 |
|  |  | >1:8 | 19/30 (63.3%) | 17/18 ^b^ (94.4%) | 1050.8 | 17/18 ^b^ (94.4%) | 809.7 |
| EV71/N0692/TW08  (B5) | A | <1:8 | 10/30 (33.3%) | 9/10 (90.0%) | 243.4 | 9/10 (90.0%) | 197.7 |
|  |  | >1:8 | 20/30 (66.7%) | 16/20 (80.0%) | 992.6 | 19/20 (95.0%) | 1027.7 |
|  | B | <1:8 | 13/30 (43.3%) | 9/11 ^a, b^ (81.8%) | 265.3 | 10/12 ^b^ (83.3%) | 183.4 |
|  |  | >1:8 | 17/30 (56.7%) | 14/17 (82.4%) | 1143.5 | 15/17 (88.2%) | 1190.1 |

Nt below detection limit (<1:8) were assigned a value of 1:4 for calculation purposes

^a^ One blood sample was misplaced and lost.

^b^ One subject withdrew from the study.

Table S3. Summary of EV71 C4 subgenotypes-specific virus neutralizing antibody titer obtained from human sera immunized with EV71 vaccine.

| EV71 virus | Group |  | | Post 1^st^-vaccination | | Post 2^nd^-vaccination | |
| --- | --- | --- | --- | --- | --- | --- | --- |
|  |  | Pre-vaccination | | >4-fold increased Nt | Neutralization titer (GMT) | >4-fold increase Nt | Neutralization titer (GMT) |
| EV71/E36  (C4A) | A | <1:8 | 8/30 (26.7%) | 7/8 (87.5%) | 98.0 | 6/8 (75.0%) | 75.1 |
|  |  | >1:8 | 22/30 (73.3%) | 18/22 (81.8%) | 930.8 | 20/22 (90.9%) | 911.8 |
|  | B | <1:8 | 10/30 (33.3%) | 6/8 ^a, b^ (75.5%) | 74.0 | 5/9 ^b^ (55.6%) | 56.0 |
|  |  | >1:8 | 20/30 (66.7%) | 15/20 (75.5%) | 995.2 | 17/20 (85.0%) | 970.4 |
| EV71/N3340/TW02  (C4B) | A | <1:8 | 18/30 (60.0%) | 5/18 (27.8%) | 10.6 | 3/18 (16.7%) | 9.7 |
|  |  | >1:8 | 12/30 (40.0%) | 5/12 (41.7%) | 40.6 | 7/12 (58.3%) | 72.9 |
|  | B | <1:8 | 19/30 (63.3%) | 3/17^a, b^ (17.6%) | 8.6 | 3/18 ^b^ (16.7%) | 10.5 |
|  |  | >1:8 | 11/30 (36.7%) | 5/11 (45.5%) | 35.9 | 6/11 (54.5%) | 93.0 |

Nt below detection limit (<1:8) were assigned a value of 1:4 for calculation purposes.

^a^ One blood sample was misplaced and lost.

^b^ One subject withdrew from study.


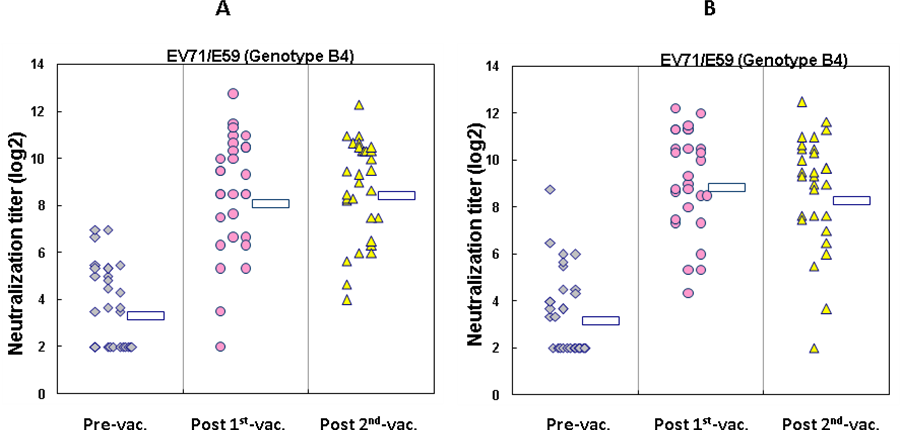


Figure S1. Virus neutralizing antibody responses of young adults immunized with EV71vac against subgenotype B4 (vaccine strain). Panels A and B show the neutralization titers of sera obtained from volunteers immunized twice with either a 5- or 10-µg dose, respectively. Thirty healthy adults were enrolled in each group. Each spot represents the neutralizing antibody titer for individual sera. The bar corresponds to the geometric mean titer (GMT) of each set of sera.
